# Roles for CEP170 in cilia function and dynein-2 assembly

**DOI:** 10.1242/jcs.261816

**Published:** 2024-05-01

**Authors:** Johannes F. Weijman, Laura Vuolo, Caroline Shak, Anna Pugnetti, Aakash G. Mukhopadhyay, Lorna R. Hodgson, Kate J. Heesom, Anthony J. Roberts, David J. Stephens

**Affiliations:** ^1^ Cell Biology Laboratories, School of Biochemistry, Faculty of Life Sciences, University of Bristol, Bristol BS8 1TD, UK; ^2^ Sir William Dunn School of Pathology, University of Oxford, Oxford OX1 3RE, UK; ^3^ Wolfson Bioimaging Facility, Faculty of Life Sciences, University Walk, University of Bristol, Bristol BS8 1TD, UK; ^4^ Proteomics Facility, Faculty of Life Sciences, University of Bristol, Bristol BS8 1TD, UK

**Keywords:** Cilia, Dynein-2, CEP170, Centrosome, Microtubule motors, Intraflagellar transport

## Abstract

Primary cilia are essential eukaryotic organelles required for signalling and secretion. Dynein-2 is a microtubule-motor protein complex and is required for ciliogenesis via its role in facilitating retrograde intraflagellar transport (IFT) from the cilia tip to the cell body. Dynein-2 must be assembled and loaded onto IFT trains for entry into cilia for this process to occur, but how dynein-2 is assembled and how it is recycled back into a cilium remain poorly understood. Here, we identify centrosomal protein of 170 kDa (CEP170) as a dynein-2-interacting protein in mammalian cells. We show that loss of CEP170 perturbs intraflagellar transport and hedgehog signalling, and alters the stability of dynein-2 holoenzyme complex. Together, our data indicate a role for CEP170 in supporting cilia function and dynein-2 assembly.

## Teaser

**Summary:** Intraflagellar transport (IFT) is required for the function of primary cilia. Centrosomal protein 170 (CEP170) interacts with the IFT motor dynein-2, and loss of CEP170 causes defects in dynein-2 assembly and cilia function.

## INTRODUCTION

Primary cilia (also called non-motile cilia) are hair-like organelles that protrude from nearly every vertebrate cell and are important for cell signalling. Mutations in cilia-related genes lead to a range of diseases, often developmental and skeletal, classified as ciliopathies (Huber and Cormier-Daire, 2012; Mill et al., 2023). Sonic hedgehog (Shh) signalling – one of the key pathways in vertebrate development – requires the primary cilium for proper signal transduction via smoothened (Smo) (Corbit et al., 2005; Huangfu et al., 2003; Mill et al., 2023). Understanding how a cilium is built and regulated is crucial for advancing our molecular understanding of ciliopathies.

At the core of the cilium is the axoneme, a structure formed of doublet-microtubules with 9-fold radial symmetry (Hall and Hehnly, 2021). The cilium is separated from the cell body by the transition zone (TZ), a region of protein fibres that restrict protein entry into the cilium (Garcia-Gonzalo et al., 2011; Garcia-Gonzalo and Reiter, 2017). At the base of the primary cilium is the basal body originating from the mother centriole. The mother centriole, along with the daughter centriole and pericentriolar material, constitute the centrosome (Gonczy and Hatzopoulos, 2019; Tischer et al., 2021). Centrioles have a 9-triplet microtubule-based structure with 9-fold radial symmetry (Gonczy and Hatzopoulos, 2019; Tischer et al., 2021). The mother centriole is distinct from the daughter in that it has additional proteinaceous structures protruding radially from the distal end, called distal appendages (DAPs) and sub-distal appendages (sDAPs) (Hall and Hehnly, 2021). DAPs are required for cilia to form as, in non-dividing cells, ciliogenesis is initiated when pre-ciliary vesicles dock with DAPs, which eventually fuse with the plasma membrane forming the ciliary membrane (Ishikawa and Marshall, 2011; Tanos et al., 2013). DAPs form a barrier between the mother centriole and axoneme (Yang et al., 2018). There is conflicting evidence surrounding the role of sDAPs in ciliogenesis. Accumulated data support a model where sDAPs assemble a stabilised microtubule network that acts in ciliary vesicle docking (Hehnly et al., 2012; Sorokin, 1962, reviewed in Hall and Hehnly, 2021).

The assembly and maintenance of a cilium requires intraflagellar transport (IFT) (Kozminski et al., 1993). IFT is an evolutionary conserved process by which material is transported in and out of the cilium and is required to overcome the barrier of the TZ and concentrate cargoes within cilia (Lechtreck, 2015). IFT requires the assembly of polymeric trains composed of the IFT-A and IFT-B protein complexes, as well as the motor protein complexes, kinesin-2 and cytoplasmic dynein-2 (hereafter referred to as dynein-2) (Pigino, 2021; Webb et al., 2020). Anterograde trafficking is powered by kinesin-2, a heterotrimeric complex formed of the kinesin-family motor proteins, KIF3A and KIF3B, and kinesin-associated protein (KAP; also known as KIFAP3) (Webb et al., 2020). When the trains reach the tip of the cilium, the trains rearrange, and subsequent retrograde transport is driven by dynein-2. The correct assembly of IFT trains is vital to ensure proper ciliary composition and signalling. As well as receptors, signalling proteins and cargo adaptors, kinesin-2 and dynein-2 motor proteins are themselves inactive cargoes during the directional transport step that they do not drive directly (Jordan et al., 2018; Toropova et al., 2017, 2019).

Dynein-2 is a large (>1 MDa) multi-subunit complex responsible for minus-end directed IFT. In humans, dynein-2 is composed of the dynein-2 heavy chain (DYNC2H1), the intermediate chains WDR60 (DYNC2I1) and WDR34 (DYNC2I2), the light intermediate chain LIC3 (DYNC2LI1), and the light chain TCTEX1D2 (DYNLT2B), together with three classes of light chain that are also found in dynein-1: Roadblock-1 or -2 (DYNLRB1 or DYNLRB2), LC8-1 or LC8-2 (DYNLL1 or DYNLL2), and TCTEX-1 or TCTEX-3 (DYNLT1 or DYNLT3) (Asante et al., 2014; Toropova et al., 2019; Vuolo et al., 2020). Mutations in all dynein-2 subunits have been shown to cause ciliopathies, often leading to defects in bone development such as Jeune asphyxiating thoracic dystrophy [JATD, also known as short-rib thoracic dysplasia (SRTD)] (Huber and Cormier-Daire, 2012; Mill et al., 2023).

Previous work has defined the interactions of WDR60 and WDR34 with other dynein-2 components and IFT proteins (Hiyamizu et al., 2023b; Shak et al., 2023; Vuolo et al., 2018). In this study, we extend that work to define the sDAP protein CEP170 as a dynein-2 interactor. We show that dynein-2 interacts with CEP170 and that cells lacking CEP170 inefficiently assemble the dynein-2 holoenzyme as well as having minor ciliary defects. Our data suggests a role for CEP170 in supporting cilia homeostasis as well as dynein-2 assembly.

## RESULTS

### CEP170, but not CEP170B, interacts with dynein-2

**Fig. 1.**
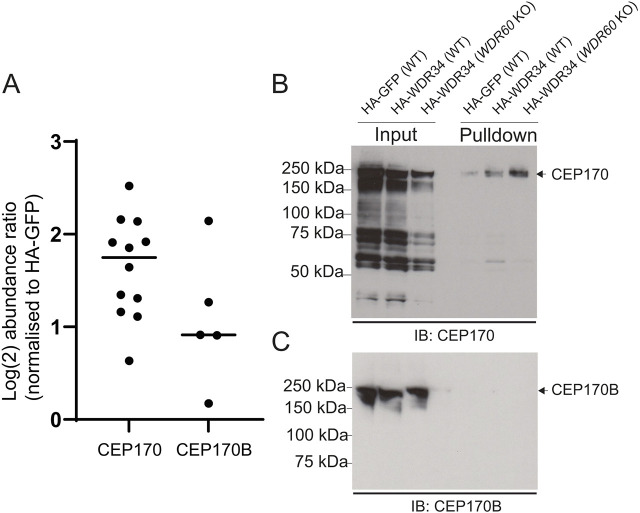
**Dynein-2 interacts with CEP170 but not CEP170B.** (A) Previous data sets from either GFP–WDR34, HA–WDR34 or HA–WDR60 interaction tandem mass tag (TMT) proteomics have reliably identified CEP170 being pulled-down with a log_2_ abundance ratio above 1. Data is presented as log_2_ abundance ratio, normalised to HA–GFP expression. A breakdown is shown in Table S1. Lines represent mean. (B,C) Co-immunoprecipitation of CEP170 (B), but not CEP170B (C), in WT RPE1 cells expressing HA–WDR34. When performed in *WDR60* KO cells more CEP170 is pulled down. Input, 5%. Blots representative of two repeats.

Previously, we have used proteomic data generated using epitope-tagged WDR60 and WDR34 (Hiyamizu et al., 2023b; Shak et al., 2023; Vuolo et al., 2018) to identify dynein-2 interacting proteins. The most consistent hit in these data sets, CEP170, was identified in 12 new and previously published tandem mass tag (TMT) datasets, 11 of which having a log_2_ abundance ratio greater than 1 (Fig. 1A; Table S1). By comparison, the related protein, CEP170B was only found in five of those datasets, and only in two with a log_2_ abundance ratio of greater than 1 (Fig. 1A; Table S1). We also previously identified CEP170 binding in non-TMT proteomic methods (Table S1) (Hiyamizu et al., 2023b). We validated the binding of CEP170 to WDR34 by co-immunoprecipitation (Fig. 1B). CEP170B has 33.4% identity with CEP170 and similar centrosomal localisation to CEP170 (Fig. S1). However, we could not detect CEP170B in co-immunoprecipitation assays with HA–WDR34 (Fig. 1C), suggesting that the interaction is specific to CEP170. Interestingly, immunoprecipitation with HA–WDR34 in the background of *WDR60* knockout (KO) cells (Vuolo et al., 2018) led to the isolation of more CEP170 (Fig. 1B). The fact that we reliably identify CEP170 in experiments with both WDR34 and WDR60 in these experiments strongly suggests an interaction with the dynein-2 holocomplex.

CEP170 is a centrosomal protein, known to locate to sDAPs on mature centrioles (Guarguaglini et al., 2005). We therefore sought to investigate whether CEP170 might have a role in cilia function.

### CEP170 KO cells can still form cilia and show defects in cilia disassembly

**Fig. 2.**
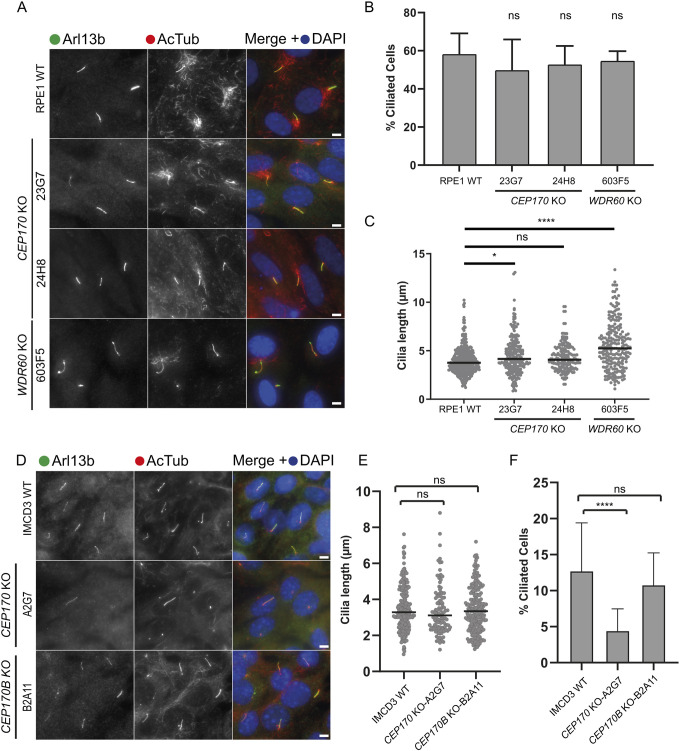
**A role of CEP170 in ciliogenesis in RPE1 and IMCD3 cells.** (A) Cilia labelled to detect Arl13b (green) and acetylated tubulin (AcTub, red) in RPE1 WT, *CEP170* KO (clones 23G7 and 24H8) and *WDR60* KO (clone 603F5) cell lines. (B) Percentage of ciliated cells [*n*=3 experiments; 636 WT, 429 *CEP170* KO (23G7), 139 *CEP170* KO (24H8), 159 *WDR60* KO (603F5) cells quantified]. (C) Cilium length in *CEP170* KO (23G7 and 24H8) and *WDR60* KO (603F5) compared with WT cells [*n*=3 experiments; 367 WT, 212 *CEP170* KO (23G7), 174 *CEP170* KO (24H8), 87 *WDR60* KO (603F5) cells quantified]. Plot in B is mean±s.d.; line in C is mean. *P=0.022; ****P<0.0001; ns, not-significant (one-way ANOVA followed by Kruskal–Wallis test). (D) Cilia were labelled to detect Arl13b (green) and acetylated tubulin (AcTub, red) in IMCD3, *CEP170* KO and *CEP170B* KO cell lines. (E) Cilium length in *CEP170* KO (clone A2G7) and *CEP170B* KO (clone B2A11) compared with WT cells [*n*=4 experiments; 213 WT, 112 *CEP170* KO (A2G7), 218 *CEP170B* KO (B2A11) cilia quantified]. Line represents mean. (F) Percentage of ciliated cells [*n*=4 experiments; 1607 WT, 2661 *CEP170* KO (A2G7), 2208 *CEP170B* KO (B2A11) cells quantified]. Plot is mean±s.d. ****P<0.0001; ns, not-significant (one-way ANOVA followed by Kruskal–Wallis test). Scale bars: 5 µm.

To further study the role of CEP170 in ciliogenesis and cilia function, we generated *CEP170* KO RPE1 (human retinal pigment epithelial cells) and mouse IMCD3 cells (Fig. S2) and performed serum-starvation induced ciliation assays (Fig. 2). *CEP170* KO RPE1 cells were able to extend cilia at the same proportion as wild-type (WT) RPE1 cells (Fig. 2A,B). In one of the RPE1 *CEP170* KO clones (23G7) there was a modest, but consistent, increase in cilia length (Fig. 2C). We also detected a modest, but statistically detectably significant, increase in cilia length in *WDR60* KO RPE1 cells in these experiments. This contrasts to our previous findings (Vuolo et al., 2018); we cannot readily explain this other than noting the intervening time and the difference in passage number of this clone. In IMCD3 cells, *CEP170* KO cells were still able to extend cilia (Fig. 2D) and the cilia were of comparable lengths to those in WT cells (Fig. 2E). However, we did observe that *CEP170* KO IMCD3 cells ciliated at a reduced proportion compared to WT IMCD3 cells (Fig. 2F).

CEP170 is recruited to sDAPs by ninein (Graser et al., 2007). In ciliated cells, ninein was still recruited to the ciliary basal body (Fig. S3A), indicating that our *CEP170* KO and *WDR60* KO cells still have sDAPs. We were able to further confirm this by electron microscopy (EM) on non-ciliated cells (Fig. S3B).

Previous work has suggested a role for CEP170 in cilia disassembly (Lamla, 2009). We tested this by re-addition of FBS to serum starved cells to trigger resorption and/or excision of primary cilia. *CEP170* KO cells remained ciliated for a much longer period than WT cells or cells lacking WDR60 (Fig. S4A,B). Live-cell imaging showed that once detected, cilia excision proceeds similarly in both WT and *CEP170* KO cells, i.e. with considerable variability in timing but visually the same with respect to GFP–Arl13B (Fig. S4C). These data confirm that CEP170 has a role in cilia disassembly.

### IFT88 accumulates at the ciliary tip in CEP170 KO cells

**Fig. 3.**
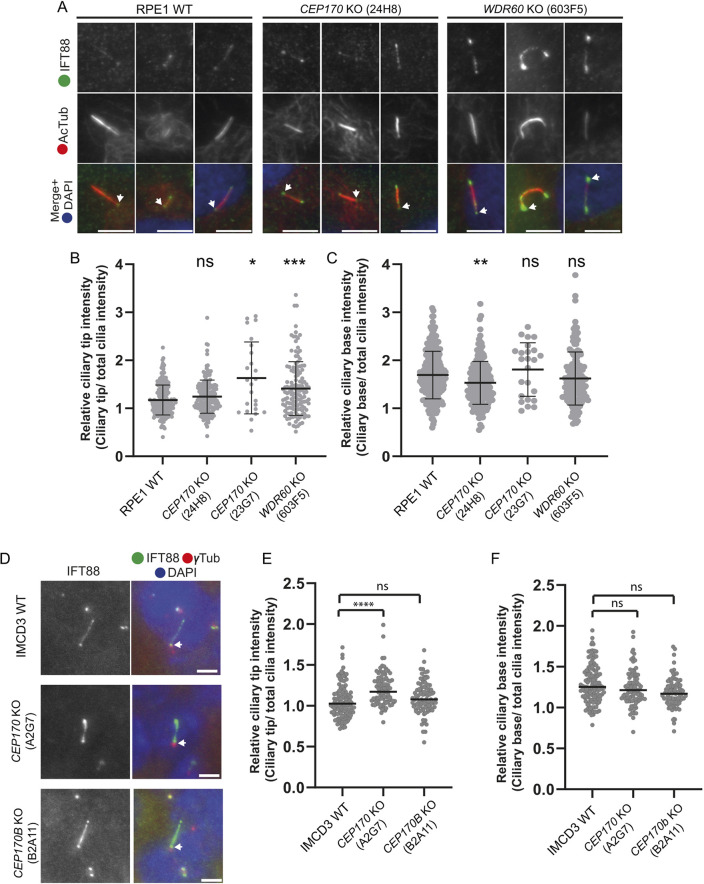
**Localisation of IFT88 in CEP170 KO cilia.** (A) Localisation of IFT88 (green) in cilia (acetylated tubulin, AcTub, red) in RPE1 WT, *CEP170* KO (clone 24H8) and *WDR60* KO (clone 603F5) cell lines. (B) Relative ciliary tip intensity of IFT88. (C) Relative ciliary base intensity of IFT88. Plots show mean±s.d. In B and C, *n*=3 experiments; 167 WT, 137 *CEP170* KO (24H8), 38 *WDR60* KO (clone 603F5) cells quantified. *P=0.0191, **P<0.003; ***P<0.0007; ns, not-significant (one-way ANOVA followed by Kruskal–Wallis test). (D) Localisation of IFT88 (green) in cilia in IMCD3 WT, *CEP170* KO (clone A2G7) and *CEP170B* KO (clone B2A11) cell lines. The basal body is marked with γ-tubulin (γTub, red). (E,F) The ImageJ plot profile tool was used to quantify the IFT88 intensity at the tip (D) and at the base (E). *n*=3 experiments; 213 IMCD3, 112 *CEP170* KO (A2G7), 218 *CEP170B* KO (B2A11) cells quantified. Bars represent means. ****P<0.0001; ns, not-significant (one-way ANOVA followed by Kruskal–Wallis test). Arrows in A and D indicate the basal body. Scale bars: 5 µm.

We next sought to address whether loss of CEP170 had any effect on IFT. IFT88 is a component of IFT-B and accumulations in ciliary tips can be used to infer defects in IFT (Hou and Witman, 2015). Serum-starved cells were fixed and labelled to detect IFT88. In *CEP170* KO RPE1 cells, we saw an increase in the proportion of cells that had IFT88 accumulations at the ciliary tip, as we see in *WDR60* KO cells (Fig. 3A,B). In IMCD3 cells, *CEP170* KO, but not *CEP170B* KO, caused an increase in the relative ciliary tip fraction of IFT88, whereas there was no difference in the relative fraction at the base (Fig. 3C–F). These data indicate that loss of CEP170 could lead to mild defects in IFT leading to an accumulation of IFT88 at the ciliary tip.

### CEP170 KO cells do not display major defects in IFT dynamics

**Fig. 4.**
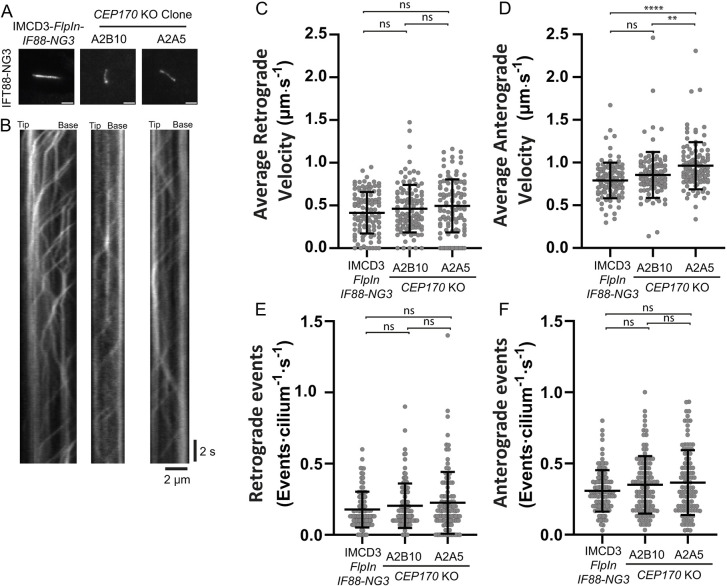
**Live TIRF imaging of IFT88-NG in CEP170 KO cells.** (A) IMCD3-*FlpIn*-*IFT88-NG3* and *CEP170* KO (clones A2B10 and A2B5) cell lines were serum starved and cilia were imaged live by TIRF microscopy (example movies are shown in Movies 1–3). Scale bars: 2 µm. (B–F) From individual cilia, kymographs were generated (B) and used to measure IFT. In the absence of a basal body marker, we assigned retrograde (C) and anterograde (D) velocities and calculate retrograde (E) and anterograde (F) events based on morphology and speed (anterograde being faster). In time-averaged images of the IFT88 channel, the base signal is typically wider than the tip signal and, in our experience, assignment based on morphology agrees with assignment based on velocity. *n*=3 experiments; 105 WT, 107 *CEP170* KO (clone A2B10), 104 (clone A2A5) cells analysed. Bars represent mean±s.d. **P=0.0019; ****P<0.0001; ns, not-significant (one-way ANOVA followed by Kruskal–Wallis test).

To further examine the IFT88 tip accumulations, we generated a *CEP170* KO (Fig. S5) in IMCD3-*FlpIn*-*IFT88-NeonGreen x3* (NG3) cells (Liew et al., 2014; Mukhopadhyay et al., 2010; Ye et al., 2018) to allow us to follow IFT in real-time using total internal reflection fluorescence (TIRF) microscopy. Live imaging (Movies 1–3) and kymograph analysis (Fig. 4A,B) was used to measure IFT velocities and events (Fig. 4C–F). We observed a slight increase in anterograde velocity for one of *CEP170* KO clone (Fig. 4D) but there was no change in retrograde velocity (Fig. 4C) nor in the number of transport events in either direction (Fig. 4E,F).

### Heightened Smo response in CEP170 KO cilia

**Fig. 5.**
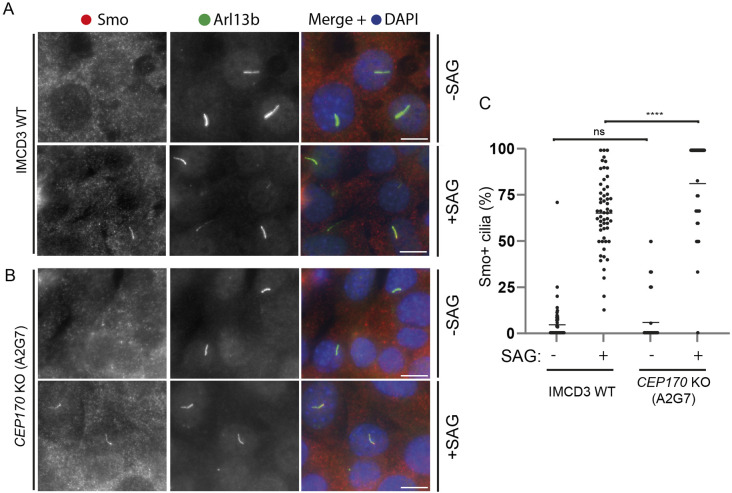
**Smo response in *CEP170* KO cells.** (A,B) IMCD3 WT (A) or *CEP170* KO (clone A2G7) (B) cells were serum starved in the absence or presence of SAG. Cells were fixed and stained for Smo (red) and the cilia marker Arl13b (green). Scale bars: 10 µm. (C) Quantification of percentage Smo-positive cilia per field of view of indicated IMCD3 cell lines in presence or absence of SAG. *n*=3 experiments; 408 IMCD3 WT −SAG, 513 WT +SAG, 123 *CEP170* KO (A2G7) −SAG, 142 *CEP170* KO (A2G7) +SAG cells counted. Bars represent means. ****P<0.0001; ns, not significant (Mann–Whitney test).

Mutations in dynein-2 have been linked to Sonic hedgehog (Shh) dysregulation (May et al., 2005). Shh signalling requires functional cilia (Breslow et al., 2018). In basal conditions, the membrane receptor Smo, part of the Shh pathway, localises to cilia but is rapidly exported by a process involving retrograde IFT. When Shh signalling is stimulated, by Shh or an agonist, such as Smo agonist (SAG), Smo instead accumulates within the cilium (Corbit et al., 2005; Hamada et al., 2018; Vuolo et al., 2018). In absence of SAG, Smo is largely absent from cilia in WT and *CEP170* KO cells (Fig. 5A,C). In the presence of SAG, we found that a greater proportion of cilia in *CEP170* KO cells accumulated Smo than in WT cells (Fig. 5B,C).

### Dynein-2 holocomplex assembly is disrupted in CEP170 KO cells

**Fig. 6.**
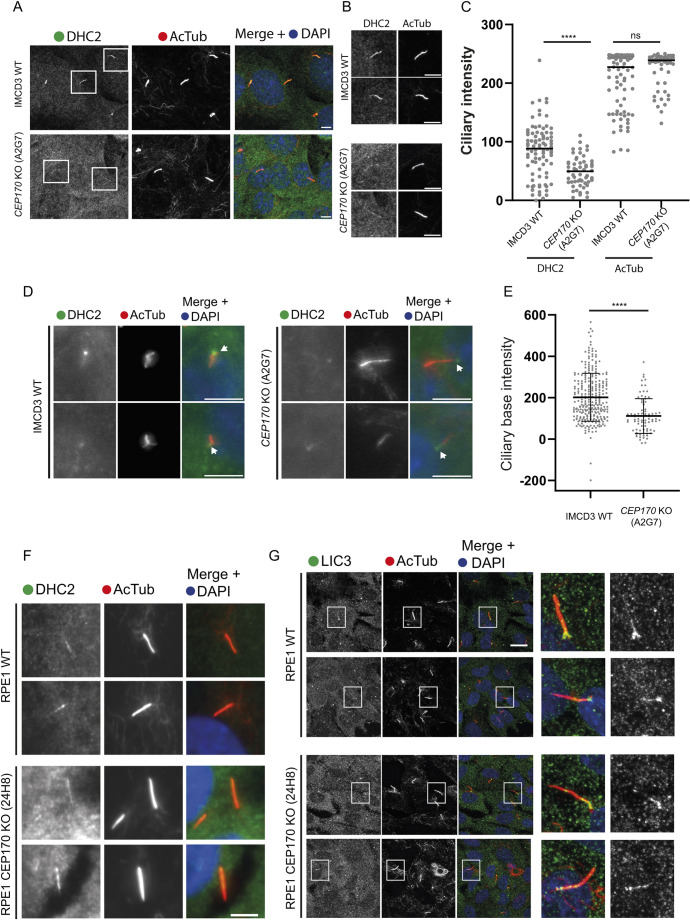
**Dynein-2 heavy chain cilia localisation in CEP170 KO cells.** (A) IMCD3 WT and *CEP170* KO (clone A2G7) cells were serum starved, fixed, and labelled for DHC2 (green) and acetylated tubulin (AcTub, red). (B) Magnified view of boxed cilia from A. (C) Intensity of the full length of the cilia was quantified. As a control, AcTub intensity was also quantified and intensities were similar in WT and KO cells [*n*=3 experiments; 85 IMCD3 WT, 55 *CEP170* KO (A2G7) cells quantified]. ****P<0.0001; ns, not significant (Mann–Whitney test). (D) Serum-starved cells were fixed and immunolabelled to detect DHC2 (green) and AcTub (red). Arrows indicate the basal body. (E) Basal body DHC2 staining intensity (normalised to background labelling adjacent to the centrosome) comparing WT and *CEP170* KO (A2G7) IMCD3 cells [*n*=3 experiments; 269 IMCD3 WT, 80 *CEP170* KO (A2G7) cells quantified]. ****P<0.0001 (Mann–Whitney test). (F) Localisation of DHC2 (green) and AcTub in WT RPE1 and CEP170 KO cells (24H8). (G) Localisation of LIC3 (DYNC2LI1, green) and AcTub in WT RPE1 and CEP170 KO cells (24H8). Images in F and G representative of three repeats. Scale bars: 5 µm.

We sought to examine the localisation of the dynein-2 heavy chain (DHC2) directly. In IMCD3 cells, immunofluorescence showed a decrease in DHC2 localisation to cilia in *CEP170* KO cells compared to WT (Fig. 6A–C). We also noted a decrease in DHC2 labelling at the base of the cilium (Fig. 6D,E). In RPE1 cells, we could detect DHC2 and LIC3 localisation to the ciliary base and within the cilium (Fig. 6F,G). Poor signal-to-noise in these experiments precluded quantification but visual inspection suggested no obvious differences in localisation of these subunits in CEP170 KO cells compared to controls. Unfortunately, currently available batches of antibodies directed against WDR34 and WDR60 are not suitable for immunofluorescence. The data from IMCD3 cells suggests that loss of CEP170 has modest but detectable effects on the localisation of dynein-2 heavy chain to cilia.

**Fig. 7.**
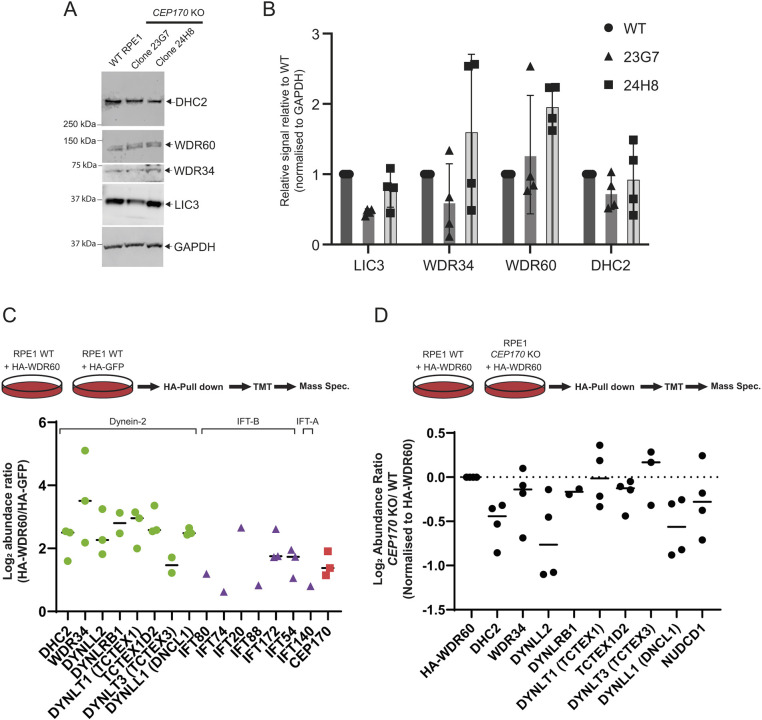
**Disruption of the dynein-2 holocomplex in CEP170 KO cells.** (A,B) Immunoblot of lysates from either WT or *CEP170* KO cells (clones 23G7 and 24H8 as indicated) showing that the indicated dynein-2 subunits are still expressed (A) and quantification (B). *n*=4. Bars represent mean±s.d. (C,D) TMT-proteomics from (C) RPE1 WT cells and (D) *CEP170* KO. Cells were transfected as indicated. C shows the abundance ratio of proteins detected in HA–WDR60 pulldowns versus HA–GFP. Dynein-2 and IFT components and CEP170 are detectable in RPE1 cells (dynein-2 subunits, green circles; IFT subunits, purple triangles; CEP170, red squares). D shows the abundance ratio of proteins detected in HA–WDR60 pulldowns in WT cells relative to *CEP170* KO cells. HA–WDR60 interaction with dynein-2 components is decreased (log_2_ relative abundance <0) in *CEP170* KO cells. Three independent experiments were performed. Data are normalised to HA–WDR60 levels. We did not detect CEP170B in either dataset (see also Table S1). Bars represent means.

As we have done previously (Vuolo et al., 2018), we used proteomic profiling to allow us to look at dynein-2 stability and assembly (Fig. 7). Immunoblotting of dynein-2-specific subunits showed that WDR34, WDR60 and DHC2 were detectable at variable levels in CEP170 KO cells but that these were not significantly different to levels in WT cells (Fig. 7A, quantified in Fig. 7B). We did see a consistent reduction in LIC3 levels in one clonal CEP170 KO line (clone 23G7) and an increase in WDR60 levels in 24H8 CEP170 KO cells. We do not have any obvious explanation for this; while it might be relevant to the loss of CEP170, this could also result from variation between clonal lines.

We then used HA-tagged WDR60 to isolate interacting proteins, the most abundant of which are typically dynein-2 subunits (Vuolo et al., 2018). In WT RPE1 cells, using HA-tagged WDR60, we could detect DHC2, the intermediate chain WDR34, dynein-2 light chains and individual components of IFT-B as well as the IFT-A component, IFT140 (Fig. 7A). We do not detect LIC3 in these data sets (we exclude detection of single peptides in our analysis); this is likely due to technical issues with either trypsinisation, separation or detection in our mass spectrometry experiments. CEP170, but not CEP170B, is also reliably co-immunoprecipitated with HA–WDR60 (Fig. 7A; Table S1) consistent with previous data (Fig. 1; Table S1).

We then repeated these same experiments in *CEP170* KO cells (Fig. 7B) to determine whether any of these interactions with HA–WDR60 were reduced or enhanced in the absence of CEP170. In CEP170 KO cells, we could still detect interaction of HA–WDR60 with DHC2, WDR34 and the known dynein-2 light chains, as well as with the chaperone NUDCD1 (which is known to be important in WD-repeat protein folding; Asante et al., 2014; Taipale et al., 2014; Vuolo et al., 2018). However, many of these interactions were detected with reduced abundance compared to control RPE1 cells. This is indicted by abundance ratios of <1 (Fig. 7B). DYNLT1 (TCTEX1) and DYNLT3 (TCTEX3) were detected at the same abundance as in controls. We do not detect CEP170B in HA–WDR60 pulldowns in either WT cells or CEP170 KO cells indicating that it cannot compensate for loss of CEP170 function in this context. These data show that loss of CEP170 results in reduced binding of several dynein-2-specific subunits to HA–WDR60. This strongly suggests a role for CEP170 in the assembly or stabilisation of the dynein-2 holocomplex.

## DISCUSSION

A functional cilium requires an intricate assembly of many multi-protein complexes including dynein-2, IFT-A and IFT-B. Our data show that CEP170 interacts with dynein-2 and promotes its assembly. Loss of CEP170 does not prevent ciliation or cause significant defects in cilia function, suggesting a possible role for CEP170 as a modulator of cilia. We do not detect CEP170B as a dynein-2 interactor, even in the absence of CEP170 itself. Furthermore, we do not find any evidence that CEP170B acts in cilia assembly or function either separately from or in synergy with CEP170. CEP170 was originally identified as a binding partner of polo-like kinase 1 (PLK1) in a yeast two-hybrid screen and shown to localise to sDAPs on mother centrioles (Guarguaglini et al., 2005). From this initial work, it was clear that CEP170 has a role in microtubule organisation, and subsequent studies have also linked it with mitosis and DNA damage responses (Qin et al., 2019; Rodriguez-Real et al., 2023; Welburn and Cheeseman, 2012; Zhang et al., 2019). CEP170 has also been shown to interact with the TZ protein RPGRIP1L (Gupta et al., 2015). CEP170 might act as a hub protein, connecting the cell cycle and cilia disassembly with dynein-2 and cilia function.

### CEP170 and sDAPs in ciliogenesis and cilia function

The mother centriole is distinct from the daughter owing to the addition of DAPs and sDAPs. The precise components of these structures are not fully defined, with new partners being identified and better microscopy techniques continuing to enable better definition of their exact location on centrioles. DAPs contain a core set of proteins, including ODF2 (Ma et al., 2023). sDAPs are comprised of ODF2, CEP128, centriolin, CCDC120, CCDC68, ninein, α- and γ-taxilin, and CEP170 (Ma et al., 2023). The consensus is that although DAPs are required for ciliogenesis, sDAPs are mostly seen to be redundant and are more important for microtubule anchoring (Hall and Hehnly, 2021; Mazo et al., 2016). Indeed, there are currently no reported ciliopathies associated with any known sDAP gene. Loss of ODF2 has been shown to affect ciliogenesis, but whether this is from their role in the sDAPs is not clear, as ODF2 is also present in DAPs (Ishikawa et al., 2005; Kashihara et al., 2019). CEP170 is located at the tip (distal) point of sDAPs, but additional non-sDAP centrosomal localisation has been observed (Guarguaglini et al., 2005; Kashihara et al., 2019; Mazo et al., 2016). Previous studies have shown that depleting either CCDC120, CCDC68 or ninein is sufficient to remove CEP170 from sDAPs, and that this does not affect ciliation (Huang et al., 2017; Mazo et al., 2016). Our results are broadly consistent with these observations; cells lacking CEP170 were still able to generate cilia of comparable lengths to WT cells. Although we observed normal length cilia in IMCD3 *CEP170* KO cells, we do note that they ciliated to a lower degree, suggesting a more general role for CEP170 and sDAPs in cilia spatial control, as previously observed (Mazo et al., 2016). However, we also see defects in cilia function, which has not been previously studied in this context. Takahara et al. (2018) reported that although KO of the IFT-A component IFT122 leads to severe ciliogenesis defects, KO of other IFT-A genes only has a minor effect on ciliogenesis but there is impaired trafficking in ciliary proteins (Hirano et al., 2017; Takahara et al., 2018). In *CEP170* KO cells, although we observed near-normal IFT velocities (Lechtreck, 2015), we did see accumulation of IFT88 at the ciliary tip, as well as impacts on SAG-dependent Smo localisation.

Cilia assembly and disassembly is linked to the cell cycle; only non-dividing cells are capable of primary cilium assembly (Breslow and Holland, 2019; Ishikawa and Marshall, 2011; Mirvis et al., 2018). Accordingly, during cell cycle progression the primary cilium must be disassembled. The processes that govern this are not fully defined and multiple mechanisms have been suggested (Breslow and Holland, 2019; Mirvis et al., 2018; Phua et al., 2017; Pugacheva et al., 2007). In our study, *CEP170* KO cells had delayed cilia disassembly, as shown previously using depletion of *CEP170* by RNA interference (Lamla, 2009). In contrast, in *WDR60* KO cells, cilia are disassembled at a rate comparable to WT cells, suggesting that the delay occurs independently of dynein-2. CEP170 might, therefore, act as a point of integration between the cell cycle and cilia disassembly, possibly via already known interactions between CEP170 and the microtubule-depolymerising kinesins KIF2A and KIF2B (Maliga et al., 2013; Miyamoto et al., 2015; Welburn and Cheeseman, 2012).

### CEP170 and dynein-2 assembly

Along with cilia defects following loss of CEP170, the main conclusion of this work is the identification of CEP170 as an interactor of dynein-2. To date, the majority of published dynein-2 interactions have only been reported in the context of IFT-A and IFT-B (Hiyamizu et al., 2023a,b; Shak et al., 2023; Toropova et al., 2019; Tsurumi et al., 2019; Vuolo et al., 2018; Zhu et al., 2021). These interactions have given us great insight into how dynein-2 interacts with IFT-A and IFT-B. Although we cannot say for certain which subunit of dynein-2 CEP170 binds to, CEP170 can be robustly detected using either WDR34 and WDR60 in immunoprecipitations. Immunoprecipitation of HA–WDR60 from *CEP170* KO cells compared to WT cells reveals that loss of CEP170 results in reduced association of HA–WDR60 with other dynein-2 subunits. These data suggest roles for CEP170 in dynein-2 holocomplex assembly. Furthermore, our data provide some limited evidence that, at least in IMCD3 cells, CEP170 has a role in maintaining DHC2 localisation at the basal body. Given the reduction in dynein-2 holocomplex it might be expected that the cilia defects would be much more severe. However, in *Chlamydomonas*, depletion of endogenous DHC2 by ∼50% does not result in any drastic defects in cilia (Reck et al., 2016). Even when DHC2 levels were more substantially reduced, cilia were still able to form. Moreover, loss of *WDR60* in nematodes and in RPE1 cells, does not ablate cilia formation or function (De-Castro et al., 2022; Vuolo et al., 2018), suggesting that cilia require only small amounts of the dynein-2 holocomplex to function. This raises questions as to why cells maintain a larger pool of dynein-2 than is needed to maintain cilia and IFT.

The dynein-2 light, light-intermediate and intermediate chains (WDR34 and WDR60) are required for dynein-2 assembly (Vuolo et al., 2018), to stabilise dimerisation of the heavy chain and to support formation of the auto-inhibited state (Perrone et al., 2003; Rompolas et al., 2007; Toropova et al., 2019). In this study, we find that immunoprecipitation of HA–WDR34 from *WDR60* KO cells captures more CEP170 than from WT cells. Considering these data together, it is possible that CEP170 interacts with intermediate chains during dynein-2 assembly and/or stabilises the complete holocomplex. CEP170 could also act in capturing dynein-2 as retrograde trains disassemble on exit from cilia (van den Hoek et al., 2022). In these ways, CEP170 could serve to enrich dynein-2 at sDAPs to promote IFT train assembly. The KIF3A subunit of the anterograde IFT motor kinesin-2 also localises to sDAPs (Kodani et al., 2013), perhaps indicating a broader role for sDAPs acting as a ‘shunting yard’ in the recruitment of components for eventual IFT train assembly.

## MATERIALS AND METHODS

Unless stated otherwise, all reagents were purchased from Sigma-Aldrich (Poole, UK).

### Plasmids

pLVX plasmids encoding HA–WDR60, HA–WDR34, HA–GFP and GFP–Arl13b were generated previously (Vuolo et al., 2018).

### Cell culture

Human telomerase-immortalised retinal pigment epithelial cells (hTERT-RPE1; ATCC CRL-4000) were grown in DMEM-F12 (Gibco) supplemented with 10% fetal bovine serum (FBS; Gibco) at 37°C with 5% CO_2_. Cells were not validated further after purchase from ATCC. *WDR60* KO RPE1 cells were generated previously (Vuolo et al., 2018). Mouse inner medullary collecting duct (IMCD-3, ATCC CRL-2123) and IMCD3-*FlpIn-IFT88-NG3* (a gift from M. Nachury, University of California San Francisco, USA) cells were grown in DMEM/F-12(HEPES) (Gibco) supplemented with 5% FBS and 100 U/ml penicillin-streptomycin at 37°C with 5% CO_2_.

### Ciliogenesis and cilia disassembly

RPE1 and IMCD3 cells were washed twice in phosphate-buffered saline (PBS) and incubated in serum-free medium for 24 h to induce ciliogenesis. For Smo experiments, confluent cells were placed in serum-free medium and treated with Shh agonist SAG (Selleckchem; from Stratech Scientific, Ely, UK; cat. no. S7779) at a final concentration of 400 nM for 24 h. A cilium disassembly assay was performed as described in Zhang et al. (2019). Specifically, cells were starved in serum-free medium for 48 h to induce cilium formation. Serum was then added back to the medium to stimulate cilium resorption. Cells were harvested at various time points for immunolabelling assays. To monitor cilia excision, RPE1 WT and CEP170 KO cells (clone 24H8) stably expressing GFP–Arl13B, were grown on imaging dishes and serum starved. Serum was added 30 min before imaging at 37°C on inverted an inverted fluoresence Leica-TIRF microscope using a 60×/1.40 oil objective. Images were collected every 2–4 min until the cilia became out of focus or photobleached.

### Genome editing

The guide RNAs (gRNA) targeting human CEP170 (RPE1 cells), mouse CEP170 and mouse CEP170B (IMCD3 cells) were designed using ‘chop chop’ software (Labun et al., 2016).

#### RPE1 cells

The gRNA sequences (5′-CAACTATGATGCGTCTA-3′; and 5′-TGGGCAGCCGTCATCGT-3′) were designed to target exon 3 and exon 7 of human CEP170. TrueCut Cas9 Protein v2 (Invitrogen) and TrueGuide Synthetic gRNA targeting CEP170 gene locus (CRISPR1123823_CR and CRISPR1123824_CR, Invitrogen) were co-transfected with TrueCut™ Cas9 Protein v2 and crRNA+tracrRNA (7.5 pmol each). into cells using Lipofectamine CRISPRMAX (Invitrogen) according to the manufacturer's instructions. After 48 h, cells were sorted, and single cells were plated in a 96-well plate (Corning). To check the *CEP170* gene, genomic DNA was extracted and the target sequences subjected to PCR to amplify targeted exons. The PCR products were cloned in the pGEM T Easy vector system (Promega) according to the manufacturer's instructions and sequenced (Eurofins Genomics and SourceBioscience). One clone was generated with with exon 3 edited (CEP170 KO 23G7) and one with exon 7 edited (CEP170 KO 24H8). Small deletions causing a frameshift were detected in both alleles (Fig. S2A,B). CEP170 KO was confirmed by immunoblotting with RPE1 WT cells (Fig. S2C).

#### IMCD3 cells

The gRNA (5′-CGAGAAATGATTTTCGT-3′) was designed to target exon 2 of mouse CEP170. Similarly, the gRNA (5′-CCACGAAGATGAGTTCACG-3′) was designed to target exon 2 of mouse CEP170B. pSpCas9(BB)-2A-GFP (PX458) (Addgene plasmid #48138, deposited by Feng Zhang; http://n2t.net/addgene:48138; RRID: Addgene_48138; Ran et al., 2013) was used as the vector to generate a gRNA for both CEP170 and CEP170B. In a well of a six-well plate (Corning), 50–60% confluent IMCD3 cells were transfected with 2.4 µg plasmid DNA, using 8.5 µl Lipofectamine 2000 transfection reagent (Thermo Fisher Scientific) in 300 µl Opti-MEM reduced serum medium (Gibco). After 48 h, GFP-positive cells were sorted and cultured for 2 weeks before single cell sorting. Single-cell sorted cells were seeded in 96-well plates (Corning) in 150 µl conditioned medium. Conditioned medium was prepared by sterile filtration of a 1:1 mixture of fresh medium, and medium removed from a flask of cells in exponential growth phase (50–70% confluent). Single-cell sorted colonies were expanded by subculture to increasingly larger culture vessels (24-well plates, 12-well plates and a T25 flask). CEP170 and CEP170B gene editing was confirmed as described for RPE1 cells. One clone was validated for CEP170 KO (CEP170 KO A2G7), with a small deletion detected in each allele causing a frameshift (Fig. S2D) and validated by immunoblotting (Fig. S2E). One clone was validated for CEP170B KO (CEP170B KO B2A11), with deletions detected in each allele causing a frameshift (Fig. S2F) and validated by immunoblotting (Fig. S2G). For IMCD3-FlpIn-IFT88-NG3 cells, two clones (both with exon 2 edited) were validated (CEP170 KO A2B10 and CEP170 KO A2A5) with changes to exon 2 (Fig. S5). Clone A2B10 has an 8-base pair deletion in one allele and a large insertion in the second allele (Fig. S5A). Clone A2A5 had deletions detected in both alleles, one causing a frameshift (Fig. S4B). Both clones were validated by immunoblotting (Fig. S5C).

### Antibodies

The antibodies used, and their dilutions for immunoblotting (IB) and immunofluorescence (IF) are as follows: mouse anti-acetylated tubulin (Sigma T6793, IF 1:2000, lot number 0000108922), rabbit anti-IFT88 (Proteintech 13967-1-AP, IF 1:300, lot 00044070), rabbit anti-DHC2 (DYNC2HC1) (ab122525, IB 1:200, lot GR247356-8), rabbit anti-DHC2 (DYNC2HC1) (ab225946, IF 1:50, lot GR3220321-11), rabbit anti-Arl13B (Proteintech 17711-1AP, IF 1:1000, lot 00076202), mouse anti-Smo (Santa Cruz Biotechnology, sc-166685, IF 1:100, lot E1721), mouse anti-GAPDH (HRP-6004, lot 21002053, 1:1000 IB), rabbit anti-CEP170 (Proteintech 27325-1-1, IF 1:500, IB 1:1000, lot 00054066), rabbit anti-CEP170B (Sigma HPA000871, IF 1:250, IB 1:300, lot r95478) mouse anti-γ-tubulin (Sigma t5326, IF 1:500, lot 10m4782v), rabbit anti-WDR34 (Novus NBP188805, IB 1:300, lot A105337), rabbit anti-WDR60 (Novus NBP1-88805, IB 1:200 lot A105337), rabbit anti-LIC3 (Proteintech 15949–1-AP, IB 1:250, lot 00007524), mouse anti-ninein (Proteintech, 67132-1-Ig, IF 1:500, lot 10008943). Alexa Fluor-conjugated secondary antibodies were from Invitrogen (A21206, A10037, A10042, A21202; IF 1:500), HRP-conjugated secondary antibodies were from Jackson ImmunoResearch (115-035-166, 111-035-144; IB 1:10,000).

### Immunofluorescence

Cells grown on 0.17 mm thick (#1.5) coverslips (Fisher Scientific, Loughborough, UK) were washed twice in PBS, and then fixed in ice-cold methanol at −20°C for 5 min. For Smo labelling, cells were fixed for 10 min at room temperature (RT) in 4% paraformaldehyde (PFA) and permeabilised with PBS containing 0.1% Triton X-100 for 5 min. For DHC2 (DYNC2HC1) labelling, cells were permeabilised with PBS containing 0.3% Triton X-100 for 10 min. Subsequently, cells were blocked with 3% bovine serum albumin (BSA) in PBS for 30 min. The coverslips were incubated with primary antibodies for 1 h, washed in PBS and then incubated with relevant secondary antibodies for 1 h. Nuclear staining was performed using DAPI (Life Technologies) at a concentration of 1 µg ml^−1^ in PBS for 3 min. Cells were then rinsed twice in PBS before mounting on glass slides (VWR) using Mowiol 4-88 mounting medium. Cells were imaged using an Olympus IX-71 widefield microscope with a 63× oil immersion objective (NA 1.4), and excitation and emission filter sets (Semrock, Rochester, NY) controlled by Volocity software (version 4.3, Perkin-Elmer, Seer Green, UK). For ciliary DYNC2HC1 (Fig. 5A,B), cells were imaged on a Leica SP5 confocal microscope system (Leica Microsystems, Milton Keynes, UK). Images were acquired as 0.5 µm *z*-stacks.

### Immunoblotting

Cells were lysed in ice-cold buffer containing 50 mM Tris-HCl pH 7.5, 150 mM NaCl, 1% Igepal and 1 mM EDTA with protease inhibitor cocktail (539137, Millipore). Samples were separated by SDS-PAGE followed by transfer to nitrocellulose membranes (Cyvita). Membranes were blocked in 5% milk in tris-buffered saline with 0.1% Tween-20 (TBST). Primary antibodies diluted in 5% milk-TBST were incubated with the membrane overnight at 4°C. Membranes were washed in TBST, incubated with secondary antibodies for 1 h, washed with TBST and detected by enhanced chemiluminescence (Promega). For Fig. 7, blots were quantified using Image Studio Lite (LI-COR). Band intensity was measured and normalised to GAPDH for each sample.

### Fluorescence intensity measurements

Quantification of fluorescence intensity was performed using average *z*-stack projections of original images in ImageJ (Schindelin et al., 2012). Local background normalised fluorescence intensity was measured at the cilary base or along the axoneme using the plot profile tool after manually tracing the axoneme in the cilary marker channel.

### Immunoprecipitation

Anti-HA immunoprecipitation was performed as previously described (Vuolo et al., 2018).

### TMT labelling and proteomic analysis

Proteins on HA–agarose beads were digested with trypsin, TMT-labelled and analysed by mass spectrometry as previously described (Vuolo et al., 2018). Three independent experiments were performed with data displayed as normalised log2 abundance ratios. The mass spectrometry proteomics data have been deposited to the ProteomeXchange Consortium via the PRIDE partner repository with the dataset identifiers PXD046827 and PXD046844.

### Live-cell IFT88 TIRF imaging

Cells were seeded (10^5^) on a 35 mm glass bottom imaging dish (MatTek) in normal medium (DMEM/F12, HEPES with 5% FBS, Gibco). The day after, cells were washed twice in PBS before being serum starved in Phenol Red-free medium (DMEM/F12, HEPES, Gibco) for 24 h. Cells were imaged at 37°C with CO_2_ on an Olympus/Abbelight SAFe360 system with two Hamamatsu Fusion sCMOS cameras, 488 nm diode laser and an 100× oil immersion objective. The pixel size was 99.7 nm. The cell surface plane was found using automated TIRF angles in the mNeonGreen channel. Abbelight NEO software was used for acquisition of movies, with frames captured every 100 ms for no longer than 1 min per movie and no more than 1 h per dish.

### IFT88 TIRF kymograph analysis

Movies of individual cilia were imported into ImageJ (Schindelin et al., 2012). Each cilium was manually traced and kymographs (in both directions) were generated using the KymographClear plug-in for ImageJ (http://www.nat.vu.nl/~erwinp/downloads.html; Prevo et al., 2015). Individual events were manually traced, and the gradient was converted to a speed (µm s^−1^) by dividing by frame length (100 ms) and multiplying by pixel size (99.7 nm). The average velocity from each cilium was plotted. Retrograde and anterograde directions were assigned based on speed (anterograde being faster) and morphology (IFT88 signal being wider at the base compared to the tip). In our experience, assignment based on morphology agrees with assignment based on velocity. The frequency was calculated by dividing the counted number of events per cilium by time. Where the slope of the trace was indistinguishable from vertical, the velocity was annotated as 0 µm s^−1^.

### Electron microscopy

Cells were grown on 35 mm dishes (Corning) before being fixed in 2.5% glutaraldehyde for 20 min and processed as previously described in Vuolo et al. (2018). Sections were imaged on an FEI (Cambridge, UK) Tecnai12 transmission electron microscope.

## Supplementary Material



10.1242/joces.261816_sup1 Supplementary information

## Data Availability

The mass spectrometry proteomics data have been deposited to the ProteomeXchange Consortium via the PRIDE partner repository with the dataset identifier PXD046827 and PXD046844.
