# Supplementary material for: Roles for CEP170 in cilia function and dynein-2 assembly
Source: J Cell Sci. 2024 May 1;137(8):jcs261816. doi: 10.1242/jcs.261816 (PMC11112123; doi:10.1242/jcs.261816)
Supplement: Supplementary information [file joces-137-261816-s1.pdf]

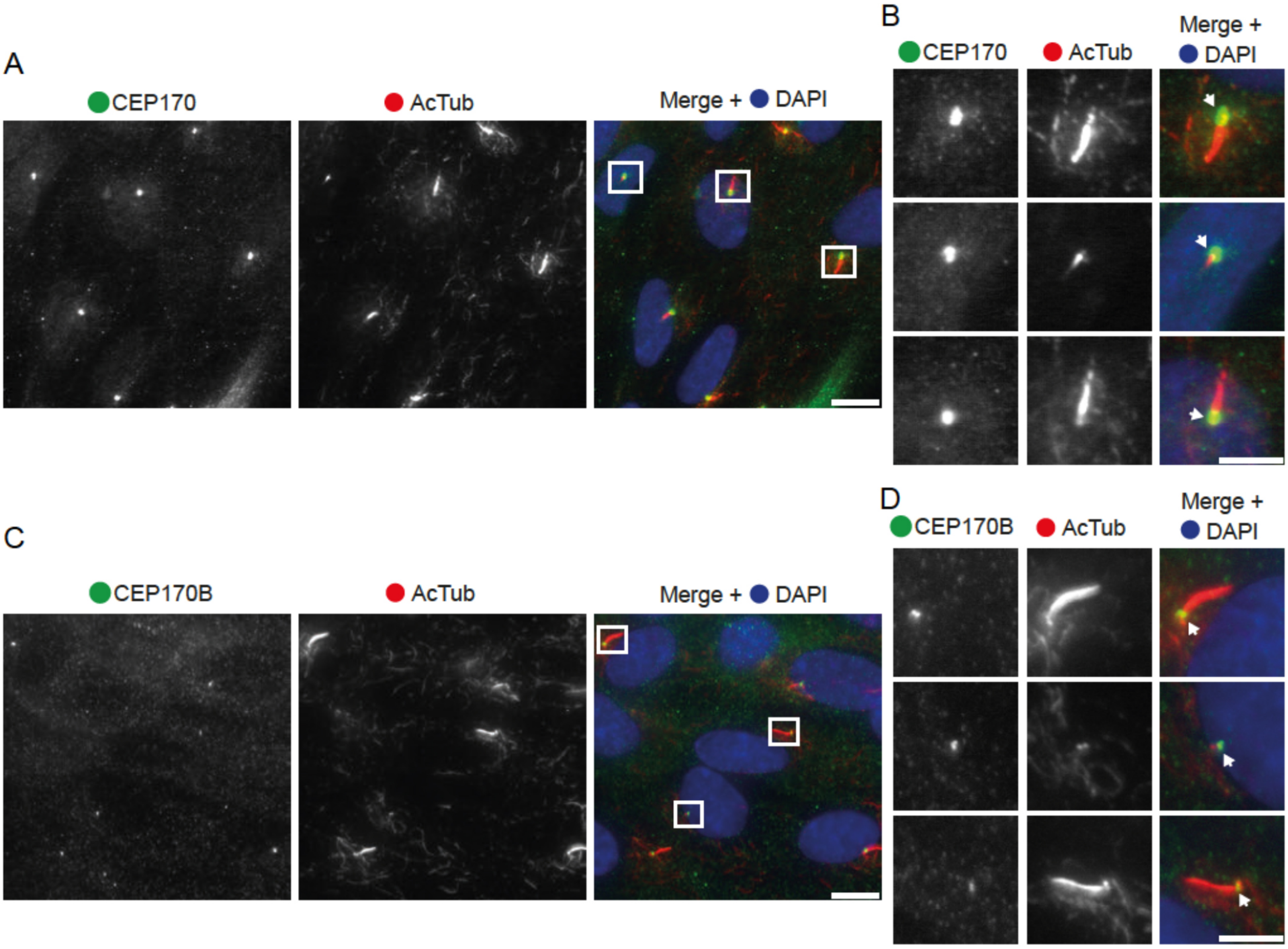

Fig. S1. Centrosomal localisation of CEP170 and CEP1708. RPE1 WT cells were serum starved and fixed. Cells were stained for either CEP170 (A and B) or CEP1708 (C and D) (green). Cilia were labelled with acetylated tubulin (AcTub, red). B and D are zoom of regions from A and C, respectively. Scale bars = 5 µm. Arrows indicate the centrosome.

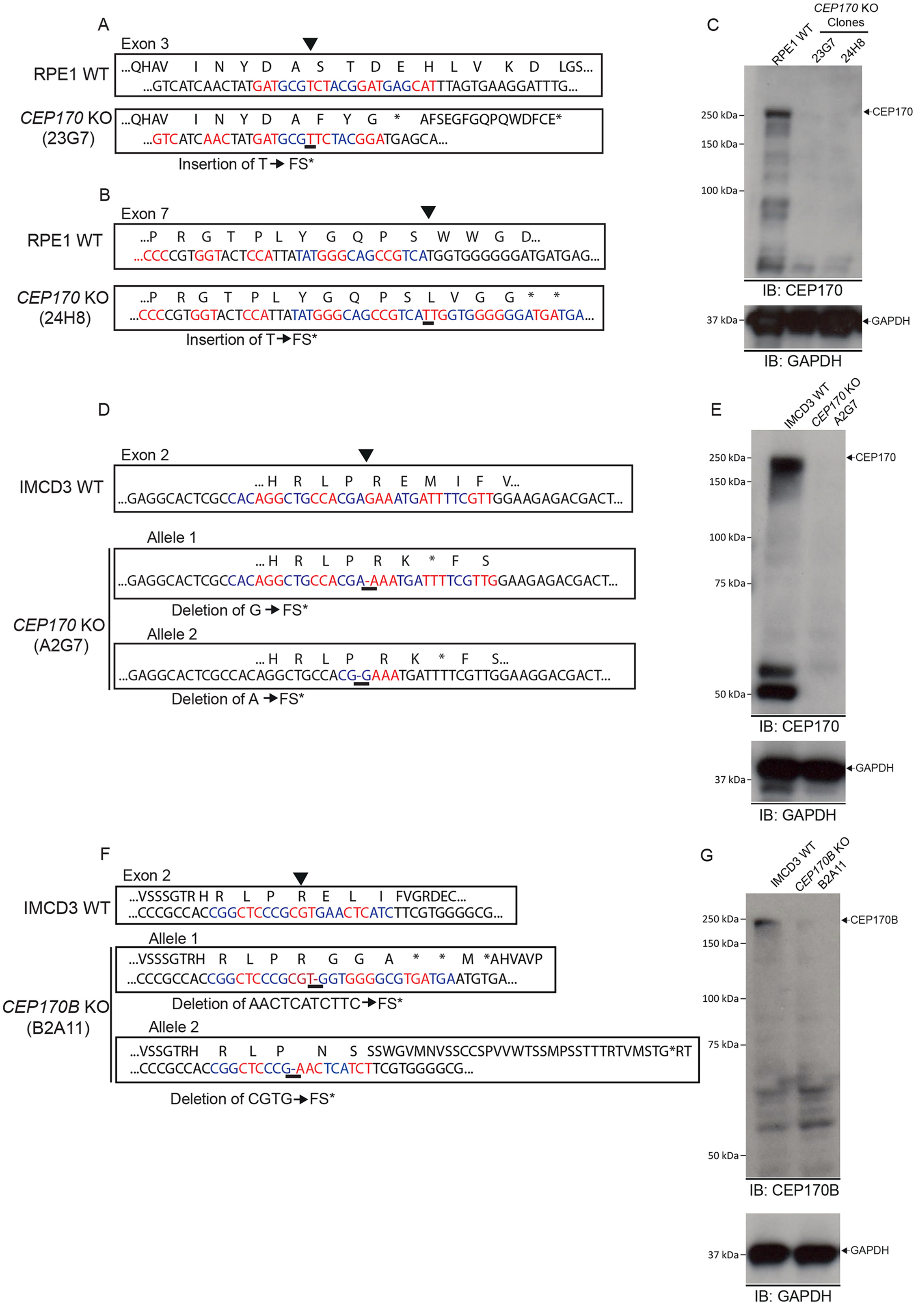

Journal of Cell Science • Supplementary information

Fig. S2. Mutation analysis for RPE1 and IMCD3 KO cell lines. (A) Alignment of human CEP170 exon 3 indicating edited region for CEP170 KO (clone 23G7) RPE1 cells. A single base pair (bp) insertion causes a frameshift and premature stop codon (FS*). (B) Alignment of human CEP170 exon 7 indicating edited region for CEP170 KO (clone 24H8) RPE1 cells. A single bp insertion causes a frameshift and premature stop codon (FS*). (C) Immunoblot (IB) on whole cell lysates from CEP170 KO clones 23G7 and 24H8 showing CEP170 is no longer present. GAPDH is shown as loading control. (D) Alignment of mouse CEP170 exon 2 indicating edited region for CEP170 KO (clone A2G7) IMCD3 cells. Different mutations were found on each allele, each a single bp deletion causing a frameshift-stop. (E) Immunoblot showing absence of CEP170 in clone A2G7. GAPDH is shown as a loading control. (F) Alignment of mouse CEP170B exon 2 indicating edited region for CEP170B KO (clone B2A11) IMCD3 cells. Different mutations were found on each allele. Allele 1 had an 11 bp deletion, allele 2 had a 4 bp deletion, each causing a frameshift-stop. (G) Immunoblot showing absence of CEP170B in clone B2A11. GAPDH is shown as a loading control. Arrowheads above alignments indicate point of modification (underlined).

Journal of Cell Science • Supplementary information

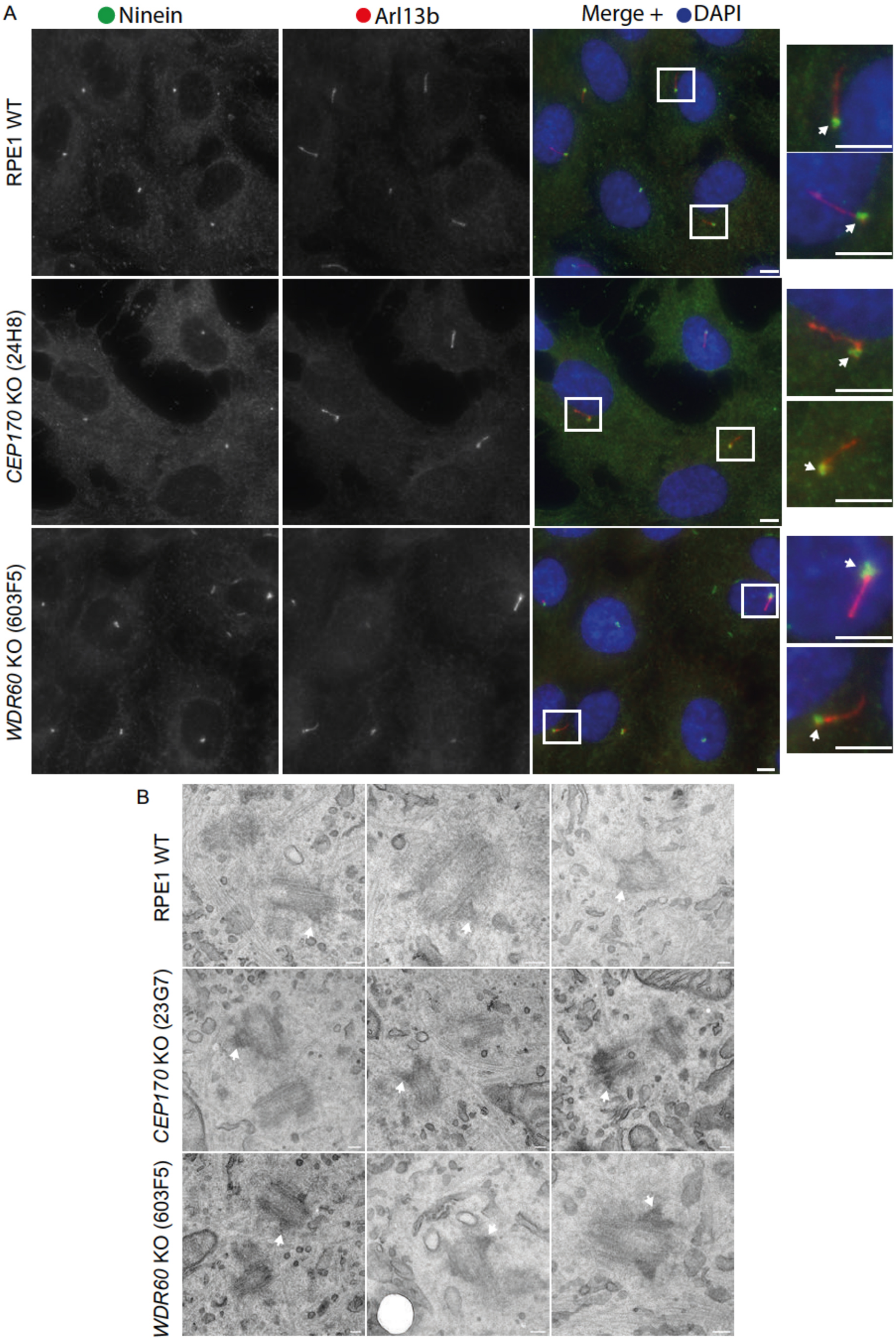
A
Ninein
Arl13b
Merge + DAPI
RPE1 WT
CEP170 KO (24H8)
WDR60 KO (603F5)
B
RPE1 WT
CEP170 KO (23G7)
WDR60 KO (603F5)

Fig. S3. sDAPs are present in CEP170 KO cells. (A) Ciliated WT RPE1, CEP170 KO (clone 24H8) and WDR60 KO (clone 603F5) cells were stained for ninein (green) and the cilia marker Arl13B (red). Ninein localises to the basal body (indicated with arrows). Scale bar = 5µm. (B) EM of non-ciliated WT RPE1, CEP170 KO (clone 23G7) cells to examine the centrioles. sDAPs can be seen on the mother centriole (indicated with arrows). Three examples are shown in each case. Scale bars = 100 nm.

Journal of Cell Science • Supplementary information

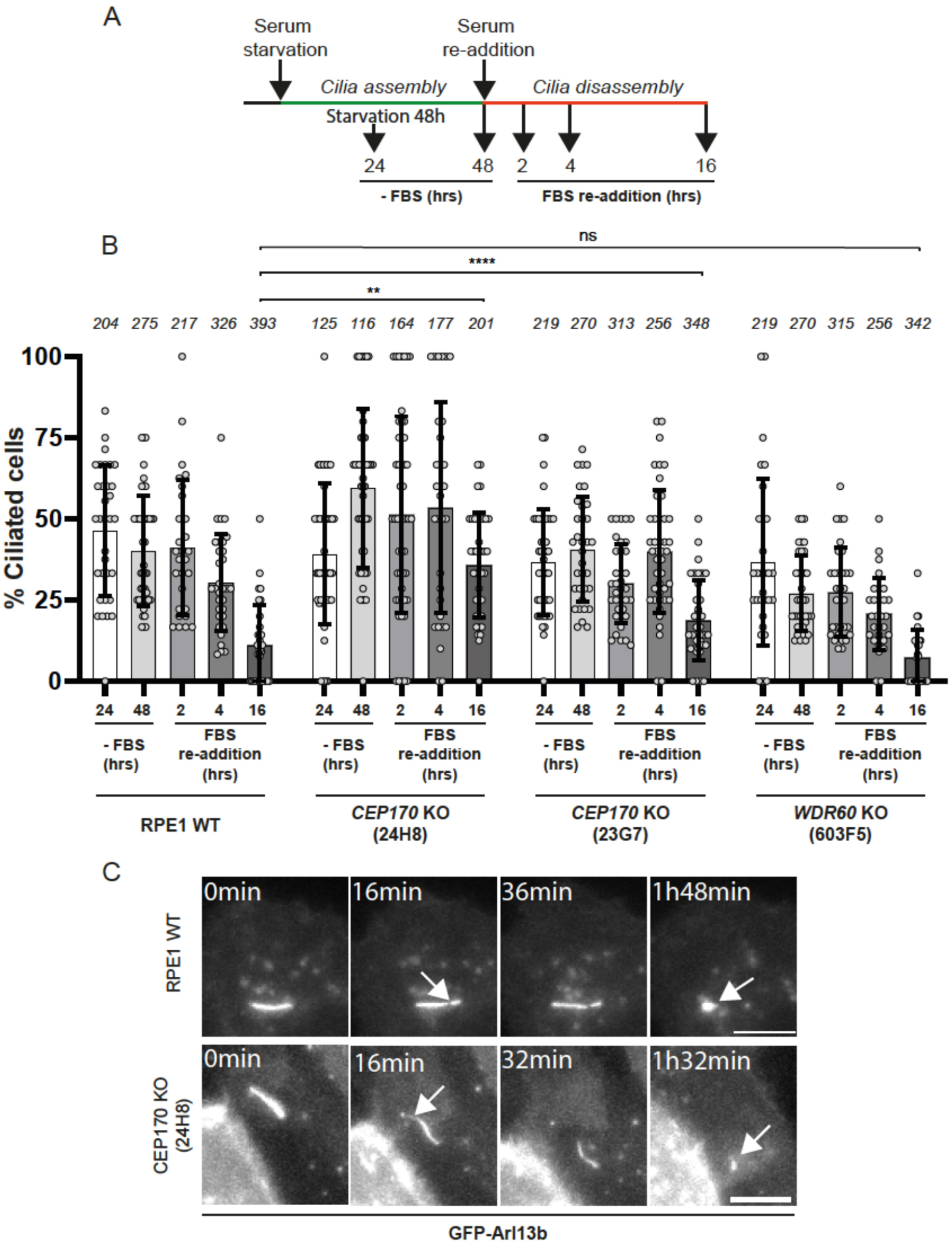

Journal of Cell Science • Supplementary information

Fig. S4. Cilia disassembly following serum re-addition. RPE1WT,WOR60 KO (603F5) and CEP170 KO (clones 24H8 and 23G7) cell lines and serum starved for 48 hours before serum was re-added and cells incubated for indicated time points (shown in A) before fixing. Cells were stained for ciliary markers (Arl13b/AcTub, as in Fig. 2) and cilia number (%) was quantified (B). Data is represented as mean ± SO. For each cell line at least three independent experiments were carried out. For each time point, the total number of cells counted is shown above in italics. Mann-Whitney two-tailed test was used to detect statistical significance at 16hrs after re-addition of serum,**- p=0.0047, **** - p<0.0001. (C) Live imaging of ciliated RPE1 WT or CEP170 KO cells stably expressing GFP-Arl13b. Serumwas re-added 30 minutes prior to imaging. Arrows indicate cilium excision points. Scale bar = 5 µm.

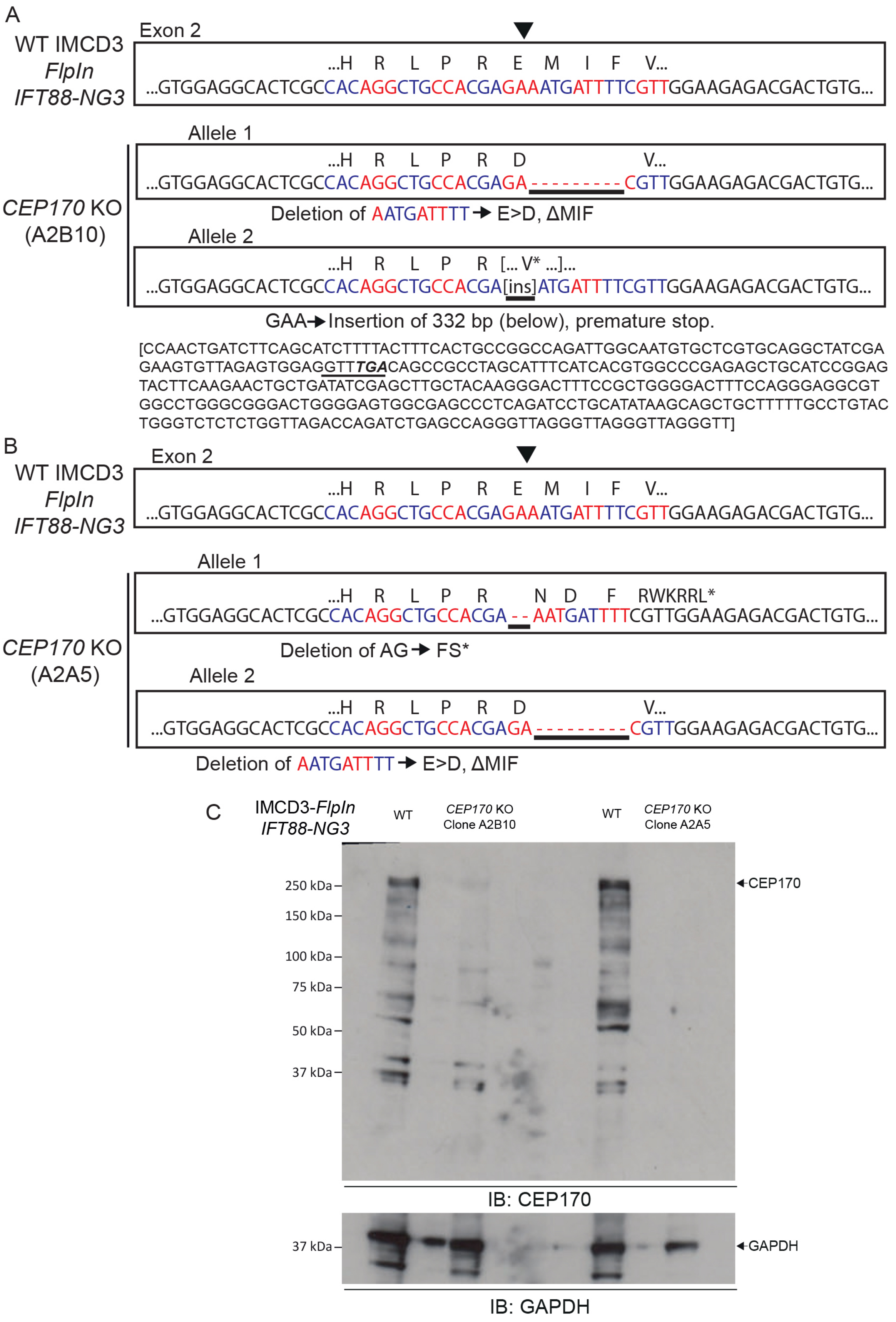

Journal of Cell Science • Supplementary information

Fig. S5. Mutation analysis for IMCD3 FlpIn-IFT88-NG3 CEP170 KO cell lines. (A) Alignment of mouse CEP170 exon 2 indicating edited region for CEP170 KO (clone A2B10) IMCD3 FlipIn-IFT88-NG3 cells. Different mutations were found on each allele. Alleles 1 had a 9 bp deletion, causing a Glu to Asp change, and triplet amino acid deletion (ΔMet-Ile-Phe). Allele 2 had a 3 bp substitution for a 332 bp region with a downstream stop codon (underlined, in-frame stop codon (TGA) is in bold italics). (B) Alignment of mouse CEP170 exon 2 indicating edited region for CEP170 KO (clone A2A5) IMCD3 FlipIn-IFT88-NG cells. Different mutations were found on each allele. Allele 1 had a 2 bp deletion causing a frameshift-stop. Allele 2 had a 9 bp deletion, causing a Glu to Asp change, and triplet amino acid deletion (ΔMet-Ile-Phe). Arrowheads above alignments indicate point of modification (underlined). (C) Immunob- lot (IB) on whole cell lysates from CEP170 KO clones A2B10 and A2A5 showing CEP170 is no longer present. GAPDH is shown as loading control.

Journal of Cell Science • Supplementary information

Table S1. Summary table of CEP170 or CEP170B found in previous dynein-2 interaction proteomics. [1]EBI Proteomics Identifications (PRIDE) database (https://www.ebi ac.uk/pride/. Perez-Riverol et al. (2022)). [2]These data sets were not isobarically labelled and so we do not report relative abundances.

Bait	Log_2 abundance ratio		Reference	EBI PRIDE Accession Code[1]
	CEP170	CEP170B		
**TMT**				
GFP-WDR34	1.161	Not found	Shak et al., 2023	PXD032758
	1.347	Not found	Shak et al., 2023	PXD032758
	1.310	Not found	Shak et al., 2023	PXD032758
	2.139	1.266	n/a	PXD046827
	2.160	Not found	n/a	PXD046827
	1.643	Not found	n/a	PXD046827
HA-WDR34	2.522	Not found	Hiyamizu et al., 2023b	PXD031151
	1.854	0.908	Vuolo et al., 2018	PXD010398
HA-WDR60	1.919	Not found	Hiyamizu et al., 2023b	PXD031151
	1.110	2.143	Hiyamizu et al., 2023b	PXD031152
	1.910	0.915	Hiyamizu et al., 2023b	PXD031153
	0.633	0.174	Hiyamizu et al., 2023b	PXD031154
	1.907	Not found	This paper (Fig. 7A)	PXD046844
	1.375	Not found	This paper (Fig. 7A)	PXD046844
	1.147	Not found	This paper (Fig. 7A)	PXD046844
**Non-TMT[2]**				
GFP-WDR34	Found	Not found	Hiyamizu et al., 2023b	PXD031157
	Found	Found	Hiyamizu et al., 2023b	PXD031158
	Found	Not found	Hiyamizu et al., 2023b	PXD031156
GFP-WDR60	Found	Found	Hiyamizu et al., 2023b	PXD031157
	Found	Found	Hiyamizu et al., 2023b	PXD031158
	Found	Not found	Hiyamizu et al., 2023b	PXD031156

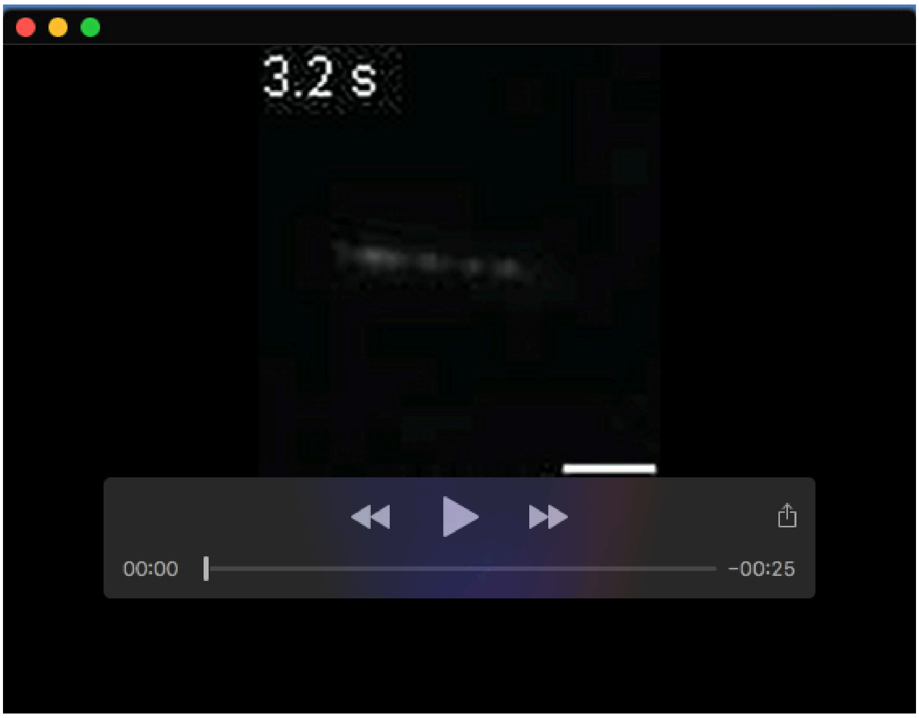

Movie 1. Representative movie from live-cell TIRF imaging. Relates to Fig. 4. IMCD3 FlipIn-IFT88-NG3. Time is indicated in the movie; playback is in real-time. Scale bar = 2 µm.

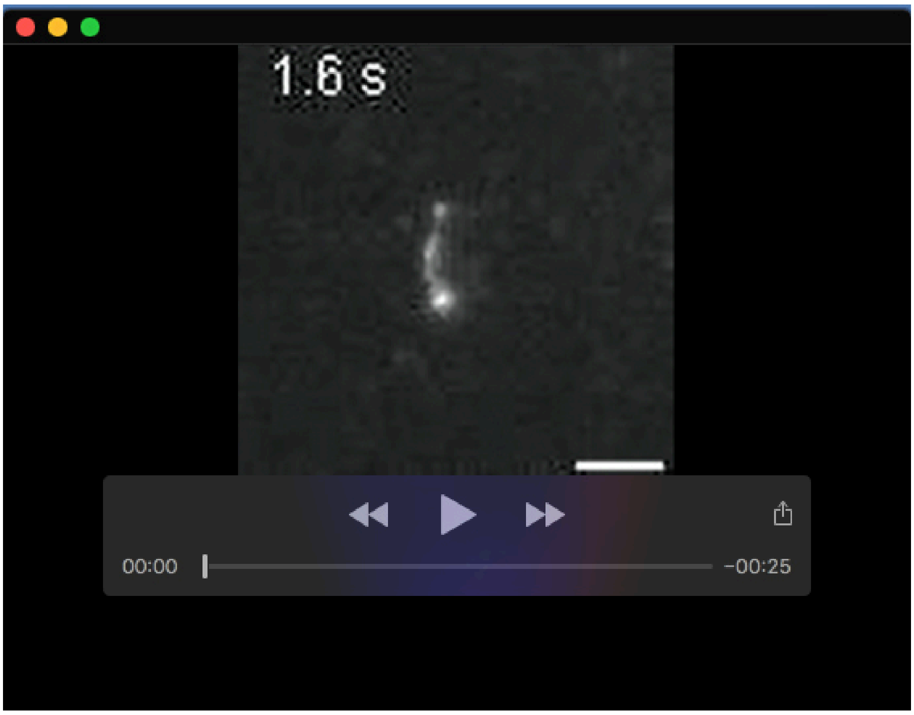

Movie 2. Representative movie from live-cell TIRF imaging Relates to Fig. 4. CEP170 KO (clone A2B10). Time is indicated in the movie; playback is in real-time. Scale bar = 2 µm.

Journal of Cell Science • Supplementary information

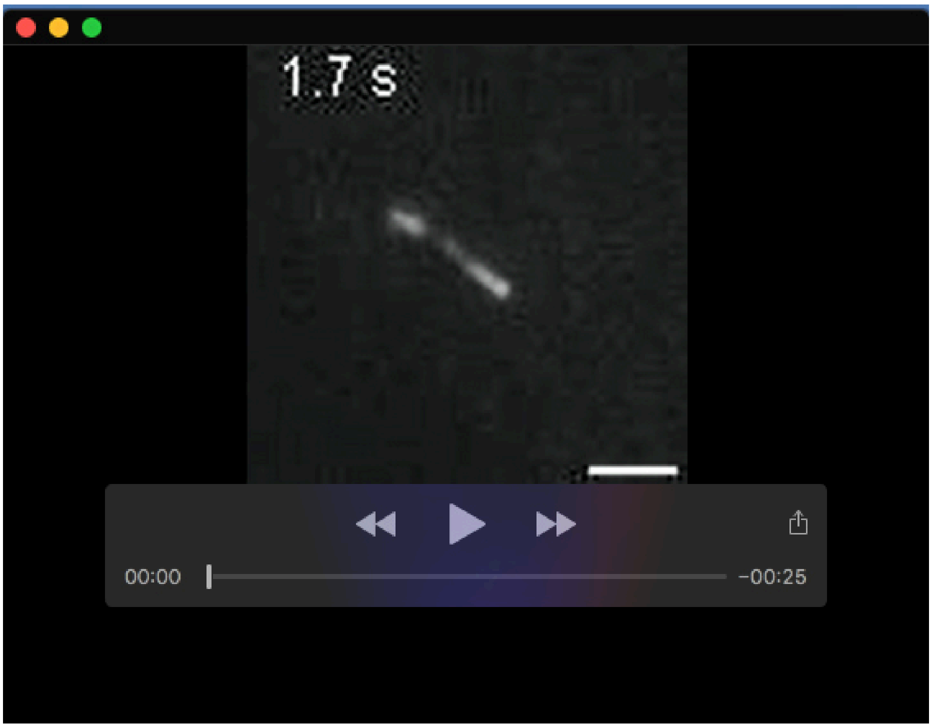

Movie 3. Representative movie from live-cell TIRF imaging. Relates to Fig. 4. CEP170 KO (clone A2B5). Time is indicated in the movie; playback is in real-time. Scale bar = 2 µm.

Journal of Cell Science • Supplementary information
