# Supplementary material for: Roles for CEP170 in cilia function and dynein-2 assembly
Source: J Cell Sci. 2024 May 1;137(8):jcs261816. doi: 10.1242/jcs.261816 (PMC11112123; doi:10.1242/jcs.261816)
Supplement: Supplementary file 1 [file joces-137-261816_review_history.pdf]

Journal of Cell Science

Roles for CEP170 in cilia function and dynein-2 assembly

Johannes F. Weijman, Laura Vuolo, Caroline Shak, Anna Pugnetti, Aakash G. Mukhopadhyay, Lorna R. Hodgson, Kate J. Heesom, Anthony J. Roberts and David J. Stephens
DOI: 10.1242/jcs.261816

Editor: Guangshuo Ou

Review timeline

Original submission:	21 November 2023
Editorial decision:	28 December 2023
First revision received:	27 February 2024
Accepted:	19 March 2024

Original submission

First decision letter

MS ID#: JOCES/2023/261816

MS TITLE: Roles for CEP170 in cilia function and dynein-2 assembly.

AUTHORS: Johannes F Weijman, Laura Vuolo, Caroline Shak, Anna Pugnetti, Aakash G Mukhopadhyay, Lorna R Hodgson, Kate J Heesom, Anthony J Roberts, and David J Stephens

ARTICLE TYPE: Research Article

We have now reached a decision on the above manuscript.

To see the reviewers' reports and a copy of this decision letter, please go to: https://submit-jcs.biologists.org and click on the 'Manuscripts with Decisions' queue in the Author Area. (Corresponding author only has access to reviews.)

As you will see, the reviewers raise a number of substantial criticisms that prevent me from accepting the paper at this stage. In particular, rescue experiments or multuple independent knockout lines are crucial for improving this work. If you think that you can deal satisfactorily with the criticisms on revision, I would be pleased to see a revised manuscript. We would then return it to the reviewers.

Please ensure that you clearly highlight all changes made in the revised manuscript. Please avoid using 'Tracked changes' in Word files as these are lost in PDF conversion.

I should be grateful if you would also provide a point-by-point response detailing how you have dealt with the points raised by the reviewers in the 'Response to Reviewers' box. Please attend to all of the reviewers' comments. If you do not agree with any of their criticisms or suggestions please explain clearly why this is so.

Reviewer 1

Advance summary and potential significance to field

© 2024. Published by The Company of Biologists under the terms of the Creative Commons Attribution License (https://creativecommons.org/licenses/by/4.0/).

The manuscript by Weijman et al. explores the potential functions of CEP170 and CEP170B in ciliogenesis, axoneme extention, IFT, dynein-2 recruitment and in the strength of WDR60's association with other subunits of the retrograde IFT motor. This research builds upon the Vuolo et al. 2018 study from the same lab, where the authors had already reported the detection of CEP170 and CEP170B when using HA-WDR34 and HA-WDR60 independently as baits for TMT proteomics. Given that both of these potential dynein-2 interactors have been implicated in the recruitment of a kinesin to mitotic spindles (PMID: 23087211), I agree with the authors that both CEP170 and CEP170B presented themselves as promising candidates for aiding in dynein-2 recruitment to the ciliary base (and even perhaps of anterograde IFT kinesins as well).
The authors started their manuscript by using co-IPs to validate the interaction of HA-WDR34 with CEP170 and CEP170B, but fail to detect the latter, concluding that only CEP170 interacts with WDR34/dynein-2. They generated CEP170 KO RPE1 and IMCD3 cell lines, and a CEP170B KO IMCD3 line to then analyse several parameters of cilia in the absence of each of the original candidates. They find that CEP170B deletion does not result in any ciliary phenotypes, while CEP170 loss affects ciliogenesis in IMCD3 (but not in RPE1), leads to a modest increase of IFT88 levels at the ciliary tip, an increase in anterograde IFT kinetics (whithout affecting retrograde IFT), a modest increase in the % of cells responsive to SAG, a significant reduction of DHC2 at the ciliary base, and a reduction in the efficiency that HA-WDR60 co-IPs other dynein-2 subunits.
While the study contributes positively to the field and benefits from the use of multiple cell types certain conclusions lack sufficient support and many corrections are necessary before the manuscript is deemed acceptable for publication.

Comments for the author

- Major concerns:

- In Figure 2, a WDR60 KO cell line is employed for comparison during the assessment of ciliogenesis and cilia length. However, contrary to what the authors have reported in Vuolo et al in 2018, WDR60 KO cilia were significantly longer than in WT, which contradicts their prior finding. The authors should determine which is the correct result and/or discuss this disparity in their manuscript.

- Also in Figure 2, the percentages of ciliated WT IMCD3 seems very low when compared with most studies in the literature, why is that? Especially considering that the example shown for WT IMCD3 cells shows more than half of them ciliated (>50%).

- In Figure 3B the % of RPE cells presenting "IFT88 staining at cilia tips" is shown. To my understanding even in controls, IFT88 labeling is normally distributed along cilia, including their tips. Therefore a qualitative + or - quantification is not very informative and can also be misleading. Another concern is that no ciliary base marker was used to distinguish it from the tip. In contrast, the quantification in D using IMCD3 cells provides a better notion of the enrichment of IFT88 at the ciliary tip in CEP170 KO cells. The authors should at least take the original data files used in Figure 2B and re-quantify it using the same approach they used for IMCD3 cells in D.

- In the kymographs of Figure 4B, the signal of IFT88-NG3 is strong at the tip of control cilia and weak at their base(!). The opposite is also true for the CEP170 KO cilia kymographs. Is the tip/base labels well positioned?
Then, this is stated in the methods section: "As we cannot tell the tip from the base, anterograde IFT was defined as being the faster of either direction."To this reviewer, this is a biased and unreasonable way to analyse IFT dynamics in mutants that may affect IFT in either direction, which can clearly lead to mistakes in the analysis. Ideally, cell lines should co-express a ciliary base marker in another channel. However, I understand that such endeavor could be unfeasible in terms of time-constraints at this point. Therefore, as an alternative, authors should at least also acknowledge this caveat in the legend of Figure 4 to be clear for readers.

- The result in Figure 6C and D is quite interesting. To avoid relying on a single antibody and further validate the result of reduced dynein-2 recruitment, the authors should repeat this experiment staining for LIC3/DYNC2LI1 as done in Vuolo et al. 2018. Alternative, they can stain for another dynein-2 subunit, for example one of the ICs.

© 2024. Published by The Company of Biologists under the terms of the Creative Commons Attribution License (https://creativecommons.org/licenses/by/4.0/).

- There are at least 2 negative intensity values in the graph of Figure 6E. I assume there were errors in the calculations. Please confirm the accuracy of all of the remaining data.

- In Figure 6F, there seem to be differences in the protein levels of WDR34. Quantifications of the bands would help to determine whether the protein levels of WDR34 are indeed altered or not in the absence of CEP170. Using a non-dynein-2 loading control will aid in the quantifications. In addition, why are WDR60 levels not shown? Assessing the levels of WDR60 becomes particularly relevant considering the authors use it as the bait for the IPs carried out in Figure 7, which they then use to infer about the state of dynein-2 assembly/integrity. In fact the experimental data used to make Figure 7 was normalized to HA-WDR60 levels (at least in one of the processing steps).

- In Figure 7, why is LIC3/DYNC2LI1 not shown? This is particularly odd given that even the smaller LCs of dynein-2 were included in the quantifications.

- One of the biggest claims/conclusions of this manuscript is that dynein-2 assembly requires CEP170 (line 40, in the abstract). This is mostly based on the data from Figure7. The co-IPs are made using only one of the complex subunits (WDR60) rather than the main DHC2 subunit itself, or at least another secondary subunit of the complex. Thus this conclusion should be rephrased to avoid overstatements. To me this data shows that CEP170 reduces the strength of the interactions of WDR60 with the remaining dynein-2 complex; authors cannot exclude whether the remaining complex remains assembled or not without additional experiments. Either provide additional data or tone down this conclusion in all of the instances it appears in the text.

- In Figure S4B, the top panel of RPGRIP1L appears to be switched between the two examples shown. It is also clear to this reviewer that some of the signal of that TZ component is spread inside cilia. If the authors decide to keep this data in the manuscript, together with the claim that the TZ is not affected quantification should be performed (at least as done in Vuolo et al. 2018).

- Based in Figure S5B, the authors state that the loss of CEP170 makes cells remain ciliated for much longer.
However, considering that the CEP170KO(24H8) ciliation levels are much higher at 48h than controls, the % of ciliated cells observed in the time points upon FBS re-adition should be normalized to the % of ciliated cells of each respective cell line just before FBS was re-added. Also clarify when do you consider that cilia disappear completely: when you only observe a spot of Arl13B(residual membrane), or when the signal is completely gone.

- Given the shared roles in the literature for CEP170 and CEP170B, could there be some redundancy between the two regarding functions in cilia? Would CEP170B be better detectable by co-IP with HA-WDR34 or HA-WDR60 in extracts lacking CEP170? One also wonders whether ciliary phenotypes would be more severe in a double KO.
However, this reviewer acknowledges that such experiments would be too time-consuming. Therefore, I just invite the authors to discuss this point further in the discussion section of the manuscript, contextualizing it further with available literature.

- Additional comments:

- Line 36 - The assertion that "dynein-2 is assembled at the base" needs to be edited. To my knowledge, there is no conclusive evidence supporting the notion that the dynein-2 complex is assembled specifically at the base of cilia, as opposed to other locations within the cytoplasm, or potentially even right upon the combined synthesis of its subunits.
- Line 146 – the authors refer to "Fig. S4" but they clearly intended to reference to data from “Fig. S6”.
- In the legend of Figure 2 is stated that lines represent the median. In the remaining Figures of the manuscript with similar data analysis state that lines represent the mean. Is there a particular reason for this or was it done in error?
- The use of “Normal” in the legend of Fig S3 is debatable: the authors should rephrase that title, toning it down to only reflect that subdistal appendages are still present in CEP170 KO. No functional assays were performed.
- The procedure used to quantify IFT frequency should be added to the methods section.

© 2024. Published by The Company of Biologists under the terms of the Creative Commons Attribution License (https://creativecommons.org/licenses/by/4.0/).

Reviewer 2

Advance summary and potential significance to field

This is a short report showing a role of CEP170 in stabilizing IFT dynein. The effects on function in tissue culture cells are slight with respect to ciliation SHH signaling, and disassembly. The strongest data are the mass spec showing the destabilization of the dynein-2 complex.

Comments for the author

Since only a single mutant was made in the two cell lines, it would be useful if another line of evidence was presented. This could include mutations in another position within the genes or multiple lines to avoid concerns about off-target effects. Certainly, the gold standard would be to perform rescue experiments with the wild-type gene with expression at the endogenous levels. In particular, it would be good to show rescue of several of the phenotypes.

Since the authors suggest a role of CEP170 in the stabilization of the dynein-2 complex, it is worth noting results found in Chlamydomonas. The amount of dynein-2 protein that is needed to build full-length cilia is very small in Chlamydomonas. The two citation below show this point. These results may be useful to explain why the phenotype of the CEP170 mutation is not obvious with respect to cilia length.

Engel BD, Ishikawa H, Wemmer KA, Geimer S, Wakabayashi K, Hirono M, Craige B Pazour GJ, Witman GB, Kamiya R, Marshall WF. The role of retrograde intraflagellar transport in flagellar assembly, maintenance, and function. J Cell Biol. 2012 Oct 1;199(1):151-67. doi: 10.1083/jcb.201206068. PMID: 23027906;
PMCID: PMC3461521.

Lin H, Nauman NP, Albee AJ, Hsu S, Dutcher SK. New mutations in flagellar motors identified by whole genome sequencing in Chlamydomonas. Cilia. 2013 Oct 30;2(1):14. doi: 10.1186/2046-2530-2-14. PMID: 24229452; PMCID: PMC4132587.

Minor points

Line 33. Primary cilia are on nearly every vertebrate cell. Blood cells lack primary cilia, adipocytes lack primary cilia although preadipocytes do have primary cilia. Toning down the nearly every statement would be good.

Line 66. IFT should replace Euro symbol FT.

Line 117 Delete can still I think a sentence like this “CEP170 KO cells form cilia” is more useful

Line 125. Consider adding Knockout to the sentence. In ciliated KNOCKOUT cells.

The sentence is confusing without the modifier

Line 139. The reference by Hou and Witman is not the first example of bulges.
Carlo Iomini showed this in 2009. PMID: 19720863; PMCID: PMC2778984.

There are many reported interactions of this protein that the authors report.
Do the knock-out support any other roles?

CEP170 appears to be mainly present in vertebrates, The lancet has the gene while many invertebrates have just the N-terminus. For example the N-terminus is in sea urchins (187 out of 1465 aa). Just as a speculation, does the N-terminus have a role?

gnomAD browser suggests that this gene does not tolerate loss of function mutations well.
(https://gnomad.broadinstitute.org/gene/ENSG00000143702?

© 2024. Published by The Company of Biologists under the terms of the Creative Commons Attribution License (https://creativecommons.org/licenses/by/4.0/).

dataset=gnomad_r4). The pLOF is 0.26. It would be useful for the authors to speculate about why LOF is not tolerated.

Reviewer 3

Advance summary and potential significance to field

In their manuscript Weijman and coauthors report on an intriguing set of experiments where they focus on the role CEP170 plays in ciliary function and structure, and in particular in IFT dynein (dis)assembly. Although the manuscript reports several interesting findings, I had here and there some difficulty assessing the novelty, importance and significance of the findings. Some times the authors are a bit brief in their reporting of the data and the drawing of conclusions could be clearer at times. Furthermore, do the data really show that CEP170 KO has a clear phenotype, in the sense that the sometimes small effects might not be due to less direct effect of CEP170 on IFT/cilia/dynein? (E.g. caused by a slightly disrupted ciliary base) I am not completely convinced, but maybe a better, more elaborate explanation of the findings might help to convince me.

Comments for the author

- Paragraph l117-136. I found it not so clear what the authors conclude from the experiments described here.
Differences between WT and KO are relatively mild. Is there a 'real' effect? The end of the paragraph is unclear, what is concluded from the rendition of FBS to serum-starved cells? And how does this connect to the experiments described above that?

- Anyway, fig 1 to fig 4 show that there is hardly an effect on IFT, ciliary shape IFT88 localization, maybe a bit on the number of ciliated cells, in the CEP170 KO cells. So is there a real phenotype?
- Fig. 5 C the distribution of the CEP170KO data looks weirdly different from WT, in that the data look very digitized (many data points have exactly the same value). Is this coincidence? Is there an explanation? This looks very weird to me... Are you sure there is no artefact?
- The discussion of the data presented in figure 7 (l 207-217) is very brief, too brief for me to be able to follow it. The final conclusion (l216-217 falls a bit out the sky.
- l 239: do the authors really show that CEP170 promotes dynein assembly? From what experiments is this conclusion drawn? Is this a conclusion from the slight decrease of dynein in the cilia (Fig 6)? Or from the changed interactions (Fig 7, which I did not completely understand).

First revision

Author response to reviewers' comments

We thank the reviewers for their careful consideration of our work and very insightful comments to improve the manuscript. We deal with each comment in turn and use blue text to indicate changes to the manuscript file.

We hope that our revisions present and explain our data in a more suitable way. We consider that our data support our conclusions and that in addition to demonstrating that dynein-2 interacts with CEP170, we show that this has clear impacts in the interaction between WDR60 and the other dynein-2 subunits. We accept that resulting outcomes in terms of cilia structure and function are limited but consider these data important in terms of understanding and would indeed be expected to be presented in such a study. We therefore hope that all reviewers will now find our work acceptable for publication in *Journal of Cell Science*.

Reviewer 1 Advance Summary and Potential Significance to Field:

The manuscript by Weijman et al. explores the potential functions of CEP170 and CEP170B in ciliogenesis, axoneme extention, IFT, dynein-2 recruitment and in the strength of WDR60's

© 2024. Published by The Company of Biologists under the terms of the Creative Commons Attribution License (https://creativecommons.org/licenses/by/4.0/).

association with other subunits of the retrograde IFT motor. This research builds upon the Vuolo et al. 2018 study from the same lab, where the authors had already reported the detection of CEP170 and CEP170B when using HA-WDR34 and HA-WDR60 independently as baits for TMT proteomics. Given that both of these potential dynein-2 interactors have been implicated in the recruitment of a kinesin to mitotic spindles (PMID: 23087211), I agree with the authors that both CEP170 and CEP170B presented themselves as promising candidates for aiding in dynein-2 recruitment to the ciliary base (and even perhaps of anterograde IFT kinesins as well).

The authors started their manuscript by using co-IPs to validate the interaction of HA-WDR34 with CEP170 and CEP170B, but fail to detect the later, concluding that only CEP170 interacts with WDR34/dynein-2. They generated CEP170 KO RPE1 and IMCD3 cell lines, and a CEP170B KO IMCD3 line to then analyse several parameters of cilia in the absence of each of the original candidates.

They find that CEP170B deletion does not result in any ciliary phenotypes, while CEP170 loss affects ciliogenesis in IMCD3 (but not in RPE1), leads to a modest increase of IFT88 levels at the ciliary trip, an increase in anterograde IFT kinetics (whithout affecting retrograde IFT), a modest increase in the % of cells responsive to SAG, a significant reduction of DHC2 at the ciliary base, and a reduction in the efficiency that HA-WDR60 co-IPs other dynein-2 subunits.

While the study contributes positively to the field and benefits from the use of multiple cell types, certain conclusions lack sufficient support and many corrections are necessary before the manuscript is deemed acceptable for publication.

We appreciate that the reviewer considers that our work contributes positively to the field. We think that the revisions made provide beter support for the conclusions and hope that this will now be acceptable for publication.

Reviewer 1 Comments for the Author: -

Major concerns:

- In Figure 2, a WDR60 KO cell line is employed for comparison during the assessment of ciliogenesis and cilia length. However, contrary to what the authors have reported in Vuolo et al in 2018, WDR60 KO cilia were significantly longer than in WT, which contradicts their prior finding. The authors should determine which is the correct result and/or discuss this disparity in their manuscript.

We have repeated these measurements to include more cilia (a more similar number to other conditions) for the WDR60 knockouts in this current study. We have also refined our analysis as these data are not normally distributed; we have now used a non-parametric test instead. It remains the case that the cilia from the WDR60 KO cells here are slightly longer - this is a modest change but is statistically detectably significant. We can only really atribute this to the fact that these data are generated ~5 years later than the original data set used in Vuolo et al and we suggest that this might be because of subtle changes in the clonal cell line used. These cells were frozen at a different time and therefore are of a different passage number. The 24H8 clone also shows some small difference in cilia length compared to controls. We consider that this supports our conclusion of small and subtle impacts on IFT. These points are now discussed in the main text.

- Also in Figure 2, the percentages of ciliated WT IMCD3 seems very low when compared with most studies in the literature, why is that? Especially considering that the example shown for WT IMCD3 cells shows more than half of them ciliated (>50%).

This is indeed correct. We chose that image to beter show the lengths as otherwise for an equivalent field of view we would only be showing ~1 cilia for that number of cells. Our IMCD3 cells are from ATCC and treated as described in the methods.

In Figure 3B the % of RPE cells presenting "IFT88 staining at cilia tips" is shown. To my understanding, even in controls, IFT88 labeling is normally distributed along cilia, including their tips. Therefore, a qualitative + or - quantification is not very informative and can also be misleading.

© 2024. Published by The Company of Biologists under the terms of the Creative Commons Attribution License (https://creativecommons.org/licenses/by/4.0/).

Another concern is that no ciliary base marker was used to distinguish it from the trip. In contrast, the quantification in D using IMCD3 cells provides a beter notion of the enrichment of IFT88 at the ciliary trip in CEP170 KO cells. The authors should at least take the original data files used in Figure 2B and re-quantify it using the same approach they used for IMCD3 cells in D.

We have now repeated this analysis as requested and include this in Figure 3 and discuss in the main text.

As with the measures of cilia length we see some subtle changes that, while statistically detectably significant, are modest. These data are again consistent with some minor impacts on IFT following loss of CEP170.

- In the kymographs of Figure 4B, the signal of IFT88-NG3 is strong at the trip of control cilia and weak at their base(!). The opposite is also true for the CEP170 KO cilia kymographs. Is the trip/base labels well positioned? Then, this is stated in the methods section: "As we cannot tell the trp from the base, anterograde IFT was defined as being the faster of either direction. "To this reviewer, this is a biased and unreasonable way to analyse IFT dynamics in mutants that may affect IFT in either direction, which can clearly lead to mistakes in the analysis.

Ideally, cell lines should co-express a ciliary base marker in another channel. However, I understand that such endeavor could be unfeasible in terms of time-constraints at this point. Therefore, as an alternative, authors should at least also acknowledge this caveat in the legend of Figure 4 to be clear for readers.

We accept that this is a limitation of the analysis. In mitigation we now have clarified our assignment of direction in the text (see ~line 150), legend and methods. In addition, we have also provided an alternative to Figure 4A (and Movie 1) that is more representative.

- The result in Figure 6C and D is quite interesting. To avoid relying on a single antibody and further validate the result of reduced dynein-2 recruitment, the authors should repeat this experiment staining for LIC3/DYNC2LI1 as done in Vuolo et al. 2018. Alternative, they can stain for another dynein-2 subunit, for example one of the ICs.

We now include data showing the localization of DHC2 and LIC3 in RPE1 cells (Figure 6G and 6H). Interestingly here, there is no difference in localization between control and CEP170 KO lines; we are not able to quantify either reliably owing to the high background labelling with each antibody. These data are now described in the text. Unfortunately, the antibody directed against LIC3 does not work in IMCD3 (mouse) cells. We have not been able to assess the impact on the localization of WDR34 or WDR60 as currently available batches of antibodies directed against intermediate chains are not suitable for immunofluorescence. Overall, our conclusion here is that both DHC2 and LIC3 can localize to the ciliary base and cilium in CEP170 knockout cells with some evidence of an impact on DHC2 localization in IMCD3 cells.

- There are at least 2 negative intensity values in the graph of Figure 6E. I assume there were errors in the calculations. Please confirm the accuracy of all of the remaining data.

This is due to normalization of the data i.e. that there is less intensity at the centrosome than in surrounding areas. This is indicated in the figure legend. We confirm the accuracy of these data.

- In Figure 6F, there seem to be differences in the protein levels of WDR34. Quantifications of the bands would help to determine whether the protein levels of WDR34 are indeed altered or not in the absence of CEP170. Using a non-dynein-2 loading control will aid in the quantifications. In addition, why are WDR60 levels not shown? Assessing the levels of WDR60 becomes particularly relevant considering the authors use it as the bait for the IPs carried out in Figure 7, which they then use to infer about the state of dynein-2 assembly/integrity. In fact, the experimental data used to make Figure 7 was normalized to HA-WDR60 levels (at least in one of the processing steps).

We have now repeated these blots and include a new version in Figure 7. Unfortunately, we always see some variability in these experiments. We assume this reflects natural biological variation but

© 2024. Published by The Company of Biologists under the terms of the Creative Commons Attribution License (https://creativecommons.org/licenses/by/4.0/).

could be compounded by poor antibodies. There is a consistent reduction in LIC3 in 23G7 CEP170 KO cells but no other consistent (or statistically detectably significant) changes across the 4 independently repeated blots.

- In Figure 7, why is LIC3/DYNC2LI1 not shown? This is particularly odd given that even the smaller LCs of dynein-2 were included in the quantifications. - One of the biggest claims/conclusions of this manuscript is that dynein-2 assembly requires CEP170 (line 40, in the abstract). This is mostly based on the data from Figure 7. The co-IPs are made using only one of the complex subunits (WDR60) rather than the main DHC2 subunit itself, or at least another secondary subunit of the complex. Thus this conclusion should be rephrased to avoid overstatements. To me this data shows that CEP170 reduces the strength of the interactions of WDR60 with the remaining dynein-2 complex; authors cannot exclude whether the remaining complex remains assembled or not without additional experiments. Either provide additional data or tone down this conclusion in all of the instances it appears in the text.

LIC3 is not routinely detected in our proteomics experiments. Where it is detected, we only count one single peptide, and our workflow excludes single peptide counts from further analysis. There are many potential reasons for this inability to detect peptides including that the protein is poorly digested, poorly separated, or poorly detected in these mass spectrometry experiments. We now expand this section and rephrase key statements. The location of WDR60 within the structure of the dynein-2 complex leads to our conclusion that it is integral to holocomplex integrity. Overall, we agree with the reviewers key phrasing that the data do indeed show reduced association of WDR60 with other dynein-2 subunits. We have amended the text in this regard at the end of the results section and reiterate this in the discussion.

- In Figure S4B, the top panel of RPGRIP1L appears to be switched between the two examples shown. It is also clear to this reviewer that some of the signal of that TZ component is spread inside cilia. If the authors decide to keep this data in the manuscript, together with the claim that the TZ is not affected, quantification should be performed (at least as done in Vuolo et al. 2018).

Apologies and thank for spoting this. Given the lack of in-depth analysis conducted here we have decided to remove these data and this figure.

- Based in Figure S5B, the authors state that the loss of CEP170 makes cells remain ciliated for much longer. However, considering that the CEP170KO(24H8) ciliation levels are much higher at 48h than controls, the %of ciliated cells observed in the time points upon FBS re-adition should be normalized to the % of ciliated cells of each respective cell line just before FBS was re-added. Also clarify when do you consider that cilia disappear completely: when you only observe a spot of Arl13B(residual membrane), or when the signal is completely gone.

We appreciate the reviewers point here. However, we are keen to avoid further processing of the data by normalization. We think the data as presented reveal important nuances such as the enhanced % cilia at 48h which would be hidden by normalization. We included these data as the validate previous work using RNAi and therefore, we consider them important comparison to previous studies. They are not however central to our conclusions as we have no evidence that this is linked to any interaction with dynein-2. We choose to retain these data but are reluctant to amend our quantification.

- Given the shared roles in the literature for CEP170 and CEP170B, could there be some redundancy between the two regarding functions in cilia? Would CEP170B be beter detectable by co-IP with HA-WDR34 or HA-WDR60 in extracts lacking CEP170?

We do not detect CEP170B in extracts lacking CEP170 (Figure 7D). There is limited evidence of shared roles or redundant functions for CEP170 and CEP170B/CEP170R in the literature. The interactions with KIF2A are different and they are differently regulated by phosphorylation in mitosis (Welburn and Cheesman, 2012). We have expanded on this in the discussion and now discuss more recent data showing that CEP170B acts at microtubule minus ends (Guan et al., 2023).

One also wonders whether ciliary phenotypes would be more severe in a double KO. However, this reviewer acknowledges that such experiments would be too time-consuming. Therefore, I just

© 2024. Published by The Company of Biologists under the terms of the Creative Commons Attribution License (https://creativecommons.org/licenses/by/4.0/).

invite the authors to discuss this point further in the discussion section of the manuscript, contextualizing it further with available literature.

We did atempt to produce some new data to address this but we were not able to validate KO of CEP170B in existing CEP170 KO lines. We do now include further discussion of potential redundancy and overlapping function point, including regarding data relating to CEP170B and KIF2A from https://europepmc.org/article/MED/37014312.

- Additional comments:

- Line 36 - The assertion that "dynein-2 is assembled at the base" needs to be edited. To my knowledge, there is no conclusive evidence supporting the notion that the dynein-2 complex is assembled specifically at the base of cilia, as opposed to other locations within the cytoplasm, or potentially even right upon the combined synthesis of its subunits.

We accept this point and have deleted this.

Line 146 – the authors refer to "Fig. S4" but they clearly intended to reference to data from “Fig. S6”.

Amended.

- In the legend of Figure 2 is stated that lines represent the median. In the remaining Figures of the manuscript with similar data analysis state that lines represent the mean. Is there a particular reason for this or was it done in error?

This should indeed refer to the mean – apologies for the error, this is now corrected this in the main MS and figure legends.

- The use of “Normal” in the legend of Fig S3 is debatable: the authors should rephrase that title, toning it down to only reflect that subdistal appendages are still present in CEP170 KO. No functional assays were performed.

We accept this and have rephrased the legend.

- The procedure used to quantify IFT frequency should be added to the methods section.

Now included.

Reviewer 2 Advance Summary and Potential Significance to Field:

This is a short report showing a role of CEP170 in stabilizing IFT dynein. The effects on function in tissue culture cells are slight with respect to ciliation, SHH signaling, and disassembly. The strongest data are the mass spec showing the destabilization of the dynein-2 complex.

Reviewer 2 Comments for the Author:

Since only a single mutant was made in the two cell lines, it would be useful if another line of evidence was presented. This could include mutations in another position within the genes or multiple lines to avoid concerns about off-target effects. Certainly, the gold standard would be to perform rescue experiments with the wild-type gene with expression at the endogenous levels. In particular, it would be good to show rescue of several of the phenotypes.

We broadly accept this point but in fact our original submission did include extensive data from two independent CEP170 KO RPE1 cell lines as well as two IMCD3 lines (one from WT IMCD3 cells) and another from mNG3-IFT88 cells).

Regarding “rescue” experiments, we agree that these would be ideal. However, we find that overexpression of CEP170 even at modest levels causes issues. The localization is not the same as detect with antibody labelling of endogenous protein. We see extensive decoration of the

© 2024. Published by The Company of Biologists under the terms of the Creative Commons Attribution License (https://creativecommons.org/licenses/by/4.0/).

microtubule network and instances where CEP170 appears to accumulate at sites of microtubule severing (possibly inducing this?). While we have sought to provide some restoration of function to our KO cells, we cannot draw clear conclusions. If we selectively score very low expressing cells, we can indeed find evidence of restoration of function (e.g. for IFT88 localization) but do not wish to include these data due to lack of robustness.

We include these data here for the benefit of the reviewers and discuss this in the main text (~line 136). This issue has already been noted elsewhere (Lamla, 2009) so we choose not to include these data in our manuscript.

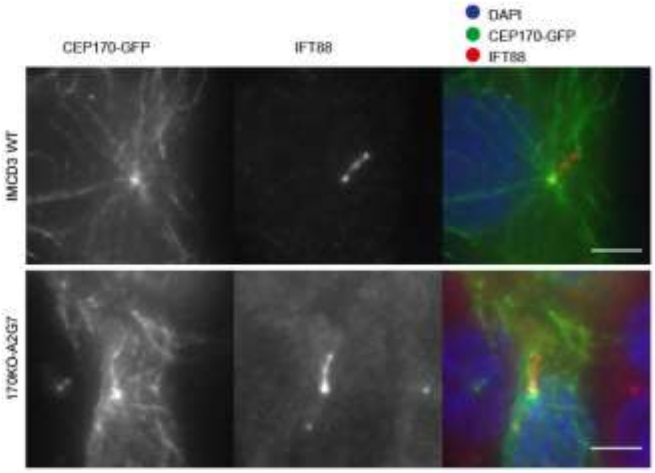

Since the authors suggest a role of CEP170 in the stabilization of the dynein-2 complex, it is worth nothng results found in Chlamydomonas. The amount of dynein-2 protein that is needed to build full-length cilia is very small in Chlamydomonas. The two citation below show this point. These results may be useful to explain why the phenotype of the CEP170 mutation is not obvious with respect to cilia length. Engel BD, Ishikawa H, Wemmer KA, Geimer S, Wakabayashi K, Hirono M, Craige B, Pazour GJ, Witman GB, Kamiya R, Marshall WF. The role of retrograde intraflagellar transport in flagellar assembly, maintenance, and function. J Cell Biol. 2012 Oct 1;199(1):151-67. doi: 10.1083/jcb.201206068. PMID: 23027906; PMCID: PMC3461521. Lin H, Nauman NP, Albee AJ, Hsu S, Dutcher SK. New mutations in flagellar motors identified by whole genome sequencing in Chlamydomonas. Cilia. 2013 Oct 30;2(1):14. doi: 10.1186/2046-2530-2-14. PMID: 24229452; PMCID: PMC4132587.

We had already discussed this to some extent in our original submission (now ~line 315+). We agree that this is an important point, and we now expand on this and include citations to this work as suggested.

Minor points

Line 33. Primary cilia are on nearly every vertebrate cell. Blood cells lack primary cilia, adipocytes lack primary cilia although preadipocytes do have primary cilia. Toning down the nearly every statement would be good.

Text amended

Line 66. IFT should replace Euro symbol FT.

Text amended

Line 117 Delete can still I think a sentence like this “CEP170 KO cells form cilia” is more useful Text amended

© 2024. Published by The Company of Biologists under the terms of the Creative Commons Attribution License (https://creativecommons.org/licenses/by/4.0/).

Line 125. Consider adding Knockout to the sentence. In ciliated KNOCKOUT cells. The sentence is confusing without the modifier

Text amended

Line 139. The reference by Hou and Witman is not the first example of bulges. Carlo Iomini showed this in 2009. PMID: 19720863; PMCID: PMC2778984. There are many reported interactions of this protein that the authors report. Do the knock-out support any other roles?

We intended to refer to data showing accumulation at the tips here. The bulges shown by Iomini might reflect the same outcome so we now include this reference. We have not investigated impacts in relation to KIF2 proteins or LK for example but do no expand on the discussion of these points.

CEP170 appears to be mainly present in vertebrates, The lancet has the gene while many invertebrates have just the N-terminus. For example the N-terminus is in sea urchins (187 out of 1465 aa). Just as a speculation, does the N- terminus have a role? gnomAD browser suggests that this gene does not tolerate loss of function mutations well. (https://gnomad.broadinstitute.org/gene/ENSG00000143702?%20dataset=gnomad_r4). The pLOF is 0.26. It would be useful for the authors to speculate about why LOF is not tolerated.

It is indeed correct that CEP170 is restricted to vertebrates but also the invertebrate chordate *Ciona*. The N- terminal region encodes the forkhead homology domain. While this can of course be analysed in isolation, we are reluctant to draw conclusions from this in the current manuscript given the clear importance of the rest of CEP170 in specifying localization and function. This does not relate to any data we present and, at worst, could mislead readers. LOF predictions are also fraught with complexity, and we consider the most likely impacts here to be on the cell division cycle through the interaction of CEP170 with polo kinase and KIF2A. Again, this is not addressed directly in our work.

Reviewer 3 Advance Summary and Potential Significance to Field:

In their manuscript Weijman and coauthors report on an intriguing set of experiments where they focus on the role CEP170 plays in ciliary function and structure, and in particular in IFT dynein (dis)assembly.

Although the manuscript reports several interesting findings, I had here and there some difficulty assessing the novelty, importance and significance of the findings.

Some times the authors are a bit brief in their reporting of the data and the drawing of conclusions could be clearer at times. Furthermore, do the data really show that CEP170 KO has a clear phenotype, in the sense that the sometimes small effects might not be due to less direct effect of CEP170 on IFT/cilia/dynein? (E.g. caused by a slightly disrupted ciliary base) I am not completely convinced, but maybe a beter, more elaborate explanation of the findings might help to convince me.

We do understand the reviewer's viewpoint here but can only present the data we obtain (even if lacking in dramatic effects). We do so for the benefit of others in the field to avoid unnecessary duplication of effort and in the hope that they can build on our findings. We consider our data fully robust and of significance.

We too were disappointed and a litle surprised not to identify more significant impacts of loss of CEP170 on cilia biology. However, our key findings are that WDR60 interacts with CEP170 and that we can define changes in dynein-2 and in IFT on perturbation of CEP170 by CRISPR KO. We can only report the data that we obtain and present them in the hope that others can build on these findings. We accept that we might have been somewhat brief in reporting our work but did so to avoid over-interpretation. We do however provide some further context and revisions throughout the text that hopefully provide greater clarity for the reviewer. These are individually detailed in our responses to other reviewers and highlighted as blue text within the revised MS file.

Reviewer 3 Comments for the Author: -

© 2024. Published by The Company of Biologists under the terms of the Creative Commons Attribution License (https://creativecommons.org/licenses/by/4.0/).

Paragraph l117-136. I found it not so clear what the authors conclude from the experiments described here. Differences between WT and KO are relatively mild. Is there a 'real' effect? The end of the paragraph is unclear, what is concluded from the rendition of FBS to serum-starved cells? And how does this connect to the experiments described above that?

We now include some further comments here and changed the section heading to reflect these data. Weconsider them important as they confirm previous work.

- Anyway, fig 1 to fig 4 show that there is hardly an effect on IFT, ciliary shape IFT88 localization, maybe a bit on the number of ciliated cells, in the CEP170 KO cells. So is there a real phenotype?

We do indeed consider these “real” phenotypes. As you will see from our responses to the other reviewers, we have tightened up these sections and provided some further images and analysis. Overall, we accept that these changes are small but report them to beter define the role of the dynein-2-CEP170 interaction. Our major outcome is clearly in terms of complex stability (Figure 7). That this doesn’t lead to dramatic changes in IFT reinforces findings from others (which we now expand on) that indicate that only relatively small quantities of dynein-2 are required for “normal” IFT. See lines 243-253.

- Fig. 5 C the distribution of the CEP170KO data looks weirdly different from WT, in that the data look very digitized (many data points have exactly the same value). Is this coincidence? Is there an explanation? This looks very weird to me... Are you sure there is no artefact?

These data report the percentage of cells with SMO positive cilia *per field of view*. These data are also taken at a lower magnification. This explains why we report so many similar values. It is also the case that many of these data are either 0 or 100%, especially for the CEP170 KO cells.

- The discussion of the data presented in figure 7 (l 207-217) is very brief, too brief for me to be able to follow it. The final conclusion (l216-217 falls a bit out the sky.

We apologise and hope that the expanded section rectifies this.

- l 239: do the authors really show that CEP170 promotes dynein assembly? From what experiments is this conclusion drawn? Is this a conclusion from the slight decrease of dynein in the cilia (Fig 6)? Or from the changed interactions (Fig 7, which I did not completely understand).

As above, we hope that the revised text (lines 182-192) beter contextualise this and explain how our data support our conclusions.

D.J.S. is no longer an editor for JCS so this conflict statement has been removed.

Second decision letter

MS ID#: JOCES/2023/261816

MS TITLE: Roles for CEP170 in cilia function and dynein-2 assembly.

AUTHORS: Johannes F Weijman, Laura Vuolo, Caroline Shak, Anna Pugnetti, Aakash G Mukhopadhyay, Lorna R Hodgson, Kate J Heesom, Anthony J Roberts, and David J Stephens

I am happy to tell you that your manuscript has been accepted for publication in Journal of Cell Science, pending standard ethics checks.

Reviewer 1

© 2024. Published by The Company of Biologists under the terms of the Creative Commons Attribution License (https://creativecommons.org/licenses/by/4.0/).

Advance summary and potential significance to field

The authors have adequately addressed all of my inquiries in the revised version.

Comments for the author

The manuscript is now suitable for publication at JCS.

Reviewer 2

Advance summary and potential significance to field

The manuscript addresses potential functions of CEP170 and CEP170B in ciliogenesis and their role in recruitment of dynein-2. Recruitment of motors to cilia is an important question and this adds to the field.

Comments for the author

The authors have addressed my concerns and it is clearer than the previous version.

Reviewer 3

Advance summary and potential significance to field

In their manuscript Weijman and coauthors report on an intriguing set of experiments where they focus on the role CEP170 plays in ciliary function and structure, and in particular in IFT dynein (dis)assembly.
Although the effects and phenotypes reported are rather weak and in that respect maybe even a bit disappointing, the results and their presentation in this revised version are sound and important to share with the community, since it will help the field in better understanding (IFT) dynein function and the role of subunits like CEP170 and others.

Comments for the author

In their revised version the authors have addressed my concerns. I think the clarity of the manuscript has improved and their comments have convinced me that it is perfectly suitable for publication in JCS in its current, revised, form.

© 2024. Published by The Company of Biologists under the terms of the Creative Commons Attribution License (https://creativecommons.org/licenses/by/4.0/).
